# Kinetics of mRNA delivery and protein translation in dendritic cells using lipid-coated PLGA nanoparticles

**DOI:** 10.1186/s12951-018-0401-y

**Published:** 2018-09-19

**Authors:** Hanzey Yasar, Alexander Biehl, Chiara De Rossi, Marcus Koch, Xabi Murgia, Brigitta Loretz, Claus-Michael Lehr

**Affiliations:** 1 Department of Drug Delivery (DDEL), Helmholtz-Institute for Pharmaceutical Research Saarland (HIPS), Helmholtz Center for Infection Research (HZI), Campus E8.1, 66123 Saarbrücken, Germany; 2 Department of Pharmacy, Saarland University, 66123 Saarbrücken, Germany; 3 Center for Bioinformatics, Saarland Informatics Campus, Saarland University, 66123 Saarbrücken, Germany; 4 INM-Leibniz Institute for New Materials, 66123 Saarbrücken, Germany

**Keywords:** mRNA, Transfection, Gene delivery, Chitosan-PLGA, Cationic lipid, Live cell imaging

## Abstract

**Background:**

Messenger RNA (mRNA) has gained remarkable attention as an alternative to DNA-based therapies in biomedical research. A variety of biodegradable nanoparticles (NPs) has been developed including lipid-based and polymer-based systems for mRNA delivery. However, both systems still lack in achieving an efficient transfection rate and a detailed understanding of the mRNA transgene expression kinetics. Therefore, quantitative analysis of the time-dependent translation behavior would provide a better understanding of mRNA’s transient nature and further aid the enhancement of appropriate carriers with the perspective to generate future precision nanomedicines with quick response to treat various diseases.

**Results:**

A lipid–polymer hybrid system complexed with mRNA was evaluated regarding its efficiency to transfect dendritic cells (DCs) by simultaneous live cell video imaging of both particle uptake and reporter gene expression. We prepared and optimized NPs consisting of poly (lactid-*co*-glycolid) (PLGA) coated with the cationic lipid 1, 2-di-*O*-octadecenyl-3-trimethylammonium propane abbreviated as LPNs. An earlier developed polymer-based delivery system (chitosan-PLGA NPs) served for comparison. Both NPs types were complexed with mRNA-mCherry at various ratios. While cellular uptake and toxicity of either NPs was comparable, LPNs showed a significantly higher transfection efficiency of ~ 80% while chitosan-PLGA NPs revealed only ~ 5%. Further kinetic analysis elicited a start of protein translation after 1 h, with a maximum after 4 h and drop of transgene expression after 48 h post-transfection, in agreement with the transient nature of mRNA.

**Conclusions:**

Charge-mediated complexation of mRNA to NPs enables efficient and fast cellular delivery and subsequent protein translation. While cellular uptake of both NP types was comparable, mRNA transgene expression was superior to polymer-based NPs when delivered by lipid–polymer NPs.

**Electronic supplementary material:**

The online version of this article (10.1186/s12951-018-0401-y) contains supplementary material, which is available to authorized users.

## Background

Messenger RNA (mRNA)-based therapeutics and vaccine strategies have gained impressive attention as an innovative, promising and alternative strategy to DNA-based therapies [1, 2]. With the unique advantages of mRNA over plasmid DNA (pDNA) preventing the requirement of nuclear entry and thereby less possibility for genomic integration, which enables transient protein translation within the cytoplasm. Thus, mRNA yields faster protein expression within the cytoplasm and serves as a favorable, effective and safe candidate with a predictable outcome for the use in biomedical research.

Viral vectors have been traditionally used as mRNA carriers due to the instability of nucleic acid macromolecules under physiological conditions [3]. Nevertheless, their use might be associated to important limitations in terms of immunologic side effects and toxicity [1]. Hence, non-viral vectors availing nanotechnological advances are in the focus of investigation to improve cellular uptake and subsequent transfection of target cells. Therefore, a variety of different biocompatible and biodegradable nanoparticles (NPs) have been developed including lipid-based [4–7] and polymer-based [8, 9] systems, featuring a cationic surface charge and thereby facilitating complexation of anionic mRNA. While these approaches rendered significant progress to overcome drawbacks resulting from mRNA-based delivery in vitro as well as in vivo [10–13], designing of suitable systems with low cytotoxicity and high transfection rate [14] remains crucial parameters and sets an indispensable precondition for mRNA-delivery. However, among all these delivery systems, a new class of nanoparticles combining the beneficial properties of both lipids and polymers termed as lipid–polymer hybrid particles (LPNs) [15] has gained momentum. Although LPNs have been commonly used for siRNA (small interfering RNA) [16–20] or pDNA [21, 22] delivery, their utility as reagents for mRNA delivery has only recently been investigated. Su et al. [11] produced a phospholipid-coated poly-(β-amino ester) (PBAE) hybrid system mediating a transfection rate of around 30% in a dendritic cell line (DC2.4 cell line), while Perche et al. [23] improved the transfection efficiency, even further, up to 60% with mannosylated histidylated lipoplexes. Besides these criteria of manufacturing safe and efficient systems, an auxiliary knowledge about the mRNA translation kinetics is important for a better understanding of mRNA’s transient course [9] for tailoring therapeutic strategies. Moreover, versatile, robust and adaptable nanocarriers are needed for scalable production. To our knowledge, the first studies evaluating the kinetics of mRNA translation were reported from Leonhardt et al. [9], in which eGFP coding mRNA complexed to Lipofectamine2000^®^ particles were used to transfect cells revealing their property as transient cargo reaching the highest protein expression rate 3 h post-transfection in A549 cells [9]. Further characterization of transgene expression kinetics has been quantified using commercially available transfection reagents, among others Stemfect^®^, in DCs [6]. Additionally, Su et al. [11] analyzed the release kinetics of surface-loaded mRNA from phospholipid coated PBAE hybrid systems and hypothesized faster release kinetics for surface adsorbed mRNA in comparison to encapsulated mRNA. Moreover, mRNA adsorbed to the particle surface showed increased stability compared to the naked one. Zhadanov et al. [24, 25] employed commercially available transfection reagents to establish a kinetic model to understand the intracellular delivery of mRNA to single cells and their subsequent release behavior within the cytoplasm. Nonetheless and to our knowledge, there is still no study available, which quantifies protein translation kinetics using tailor-made mRNA nanocarriers.

**Fig. 1:**
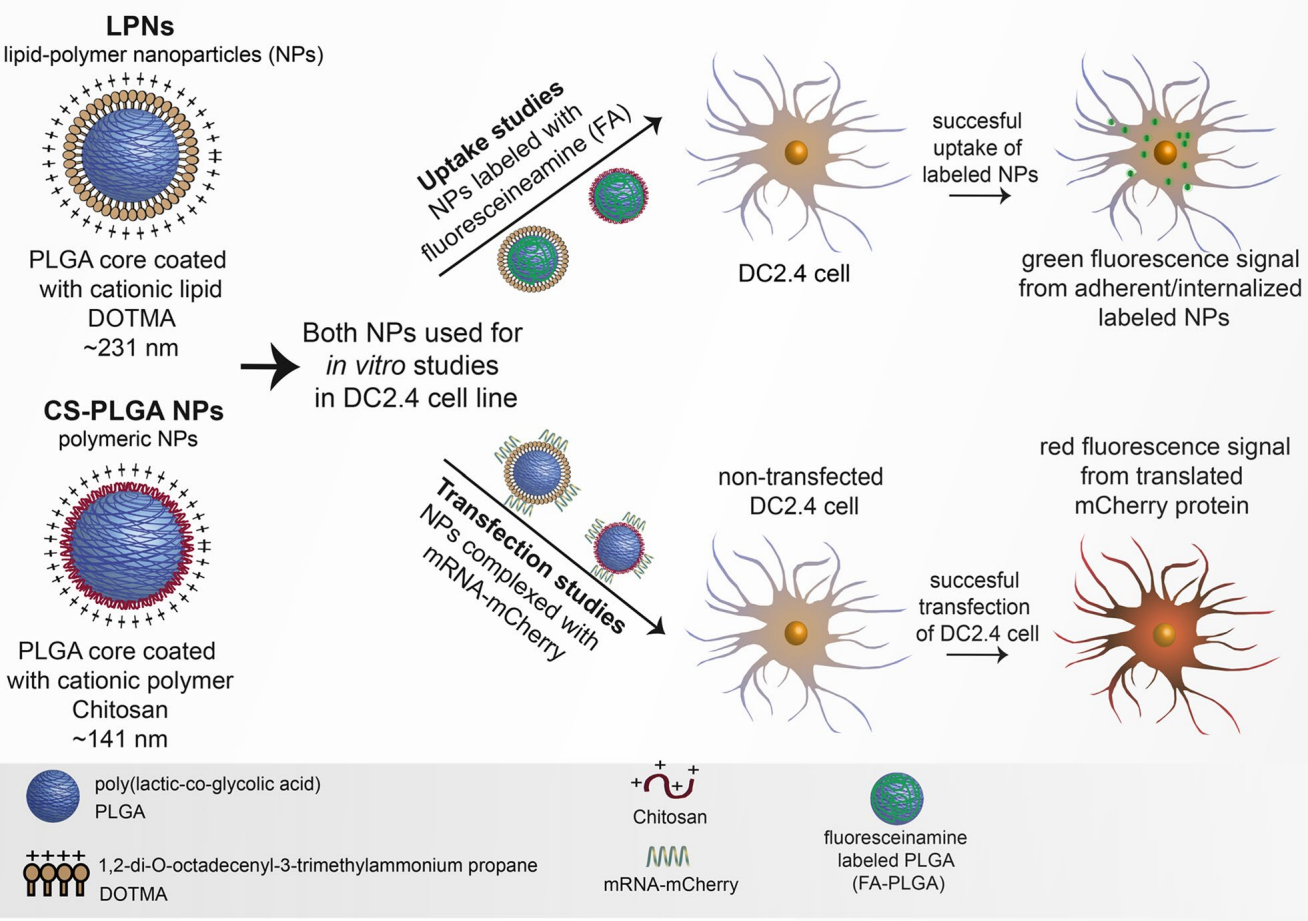
Schematic illustration of all nanoparticles used in this study. Both, LPNs and CS-PLGA NPs either complexed with mRNA or/and labeled with fluoresceinamine are used to quantify their uptake behavior and transfection efficiency in a dendritic cell line (DC2.4)

Therefore, the aim of the study was to understand and evaluate the time-dependent internalization behavior of mRNA in vitro in DCs using two different types of tailor-made nanocarriers as illustrated in Fig. 1. DCs represent potent antigen presenting cells (APCs) and are the most essential targets for mRNA vaccines [26]. Therefore, it is also the purpose of this study to transfer the gained knowledge for future NP-based vaccination strategies. Hence, (i) we produced core–shell structured lipid–polymer hybrid nanoparticles (LPNs), co-formulated with the biodegradable and biocompatible polymer poly (lactid-*co*-glycolid) (PLGA) as the core surrounded by the well-known cationic lipid 1,2-di-*O*-octadecenyl-3-trimethylammonium propane (DOTMA) [27]. LPNs were then complexed with mRNA-mCherry, which provided a reliable read-out to evaluate transfection efficiency. Additionally to that, (ii) the internalization kinetics of LPNs to DCs was characterized and systematically compared with a well-established delivery system consisting of a cationic polymer chitosan coating PLGA [10, 28] (CS-PLGA NPs). mCherry encoding mRNA was applied as a proof of concept model for facile observation and post-loaded onto the nanoparticles to achieve the expected fast, desired release kinetics and hence protein expression. Additionally to that, we monitored real-time transfection by, (iii) applying live cell video imaging in order to simultaneously analyze both the time-dependent uptake of fluorescently labeled, mRNA-loaded LPNs and the translation of the mCherry protein within DCs.

## Experimental methods

### Preparation and characterization of blank lipid-PLGA (LPNs) and chitosan-PLGA (CS-PLGA) nanoparticles

Both types of nanoparticles used in this study have been produced in the same way using a modified double-emulsion method as described previously [28]. PLGA (50:50; Resomer RG 503H, Evonik Industries AG, Darmstadt, Germany) served as the core polymer and 1,2-di-*O*-octadecenyl-3-trimethylammonium propane (DOTMA) or chitosan as the cationic surface layer. All deployed organic solvents in the experiments were purchased from Sigma-Aldrich (Darmstadt, Germany).

Lipid-PLGA NPs (LPNs) were produced by taking a protocol described by Jensen et al. as starting point [17], but replacing the cationic lipid 1,2-dioleoyl-3-trimethylammonium-propane (DOTAP) by DOTMA and few further modifications.

Briefly, a solution composed of 125 μL of DOTMA (13 mg/mL; Avanti polar lipids, INC, AL, USA) and 250 μL of PLGA (30 mg/mL) was prepared in chloroform and mixed thoroughly. A volume of 250 μL of milli-Q water (Merck Millipore, Billerica, MA) was then added to the DOTMA:PLGA organic phase followed by a subsequent sonication with ultrasound (Branson Ultrasonic Corporation, USA) at 30% amplitude for 30 s enabling the primary w/o emulsion. Immediately afterwards, 1 mL of a 2% (w/v) polyvinyl alcohol (PVA, Mowiol^®^ 4-88, Sigma-Aldrich, Darmstadt, Germany) solution was applied to the primary emulsion and sonicated under the same settings resulting in the secondary w/o/w emulsion. Further, 5 mL of the PVA solution were then added to the secondary emulsion under continuous stirring overnight to allow organic solvent evaporation. Resulting DOTMA coated PLGA NPs (2.15 mg/mL) were purified using a dialysis membrane (MWCO 1 kDa, Spectrum Labs, CA, USA).

Chitosan-PLGA NPs were prepared according to [28, 29]. Briefly, 0.2% (w/v) of chitosan solution was prepared by dissolving Protasan UP CL 113 (FMC Biopolymer AS NovaMatrix, Sandvika, Norway) in a 2% (w/v) PVA solution. Afterwards, 50 mg of PLGA was dissolved in 2 mL of ethyl acetate and 400 μL of milli-Q water was then added to the PLGA organic phase followed by a subsequent sonication to obtain the primary w/o emulsion. The chitosan-PVA solution was immediately afterwards added to the w/o emulsion and sonicated once again resulting in the secondary w/o/w emulsion. A volume of 20 mL of milli-Q water was additionally added to the w/o/w emulsion to allow organic solvent evaporation with a further purification of resulting chitosan coated PLGA NPs (2 mg/mL) by centrifugation at 15,000*g* for 15 min. To examine the cellular localization of the NPs, fluorescently labeled NPs were produced using fluoresceinamine (FA; Sigma-Aldrich, Darmstadt, Germany) covalently coupled to PLGA [30] and simply substituted against PLGA in the above mentioned production-procedure resulting in FA-LPNs and CS-FA-PLGA NPs.

The physicochemical properties of all NPs were characterized by dynamic light scattering (DLS; Zetasizer Nano, Malvern Instruments, UK) to attain their hydrodynamic size (referred to the hydrodynamic diameter), polydispersity index (PDI) and ζ-potential. All values obtained by DLS are the mean values of the peak intensity distribution. All samples were measured at least in four different batches and results are shown as the mean ± standard deviation (SD).

### Structural characterization of blank nanoparticles using SEM, TEM and Cryo-TEM

The morphological appearance of all nanoparticles was visualized using a variety of different microscopical methods including conventional Scanning Electron Microscopy (SEM, EVO HD15, Zeiss, Germany) and Transmission Electron Microscopy (TEM, JEM 2011, JEOL, St Andrews, UK). Before TEM-visualization, 10 μL of each NP dispersion was applied on a carbon coated copper grid (type S160-4 from Plano GmbH, Wetzlar, Germany) and the excess solution was removed after 10 min incubation time. In order to improve the contrast of the TEM-images, adhered NPs on the copper grid were in another experimental setting further stained with 0.5% (w/v) phosphotungstic acid solution (PTA; Sigma-Aldrich, Darmstadt, Germany) according to our previous studies described in Yasar et al. [31]. For SEM visualization, the copper grid with applied NPs were then placed onto a carbon disc and gold-sputtered. For cryo-TEM investigations 3 µL of the NPs solution were placed onto a holey carbon film (type S147-4 from Plano GmbH, Wetzlar, Germany), plotted for 2 s to a thin film and plunged into liquid ethane using a cryo plunge 3 system from Gatan (Pleasanton, CA, USA) operating at T = 108 K. The frozen samples were transferred under liquid nitrogen to a cryo-TEM sample holder (Gatan model 914) and imaged in bright-field low-dose mode (JEOL JEM-2100) at T = 100 K and 200 kV accelerating voltage.

### Physical stability of LPNs and CS-PLGA nanoparticles under physiological conditions

The physical stability of blank LPNs was tested over a time course of 62 days upon storage at 4 °C and room temperature. Additionally, the stability of both blank NPs was characterized in Hanks’s Balanced Salt Solution (HBSS buffer, pH 7.4) and in Dulbecco’s Modified Eagle Medium (DMEM; Thermo Fisher Scientific, Darmstadt, Germany) with and without 10% fetal calf serum (FCS; Sigma-Aldrich, Darmstadt, Germany) at different time-points in order to find the best conditions for in vitro cell culture studies. Briefly, 0.215 mg/100 μL of blank LPNs and 0.2 mg/100 μL of CS-PLGA NPs were mixed with 800 μL of appropriate medium. The samples were incubated at 37 °C with 5% CO_2_ under slightly shaking for 2 h, 4 h, and 24 h. Immediately afterwards, the hydrodynamic size, PDI, and ζ-potential were measured from three independent samples and results are presented as mean ± SD.

### Preparation of mRNA-mCherry loaded NPs

mRNA-mCherry (CleanCap™ mCherry mRNA (5moU); TriLink BioTechnologies LLC, CA, USA) was loaded at different ratios to both LPNs and CS-PLGA NPs to evaluate their potential as efficient mRNA delivery systems. Thus, the anionic mRNA was loaded onto the surface of both cationic NPs (following our previous protocol described in Yasar et al. [31]) using mRNA:NPs weight ratios of 1:10, 1:20 and 1:30. A volume of 1 μg/μL mRNA-mCherry was mixed with an appropriate amount of each NPs and further incubated at room temperature for 1 h. This carried out in mRNA complexed NPs (mRNA:LPNs and mRNA:CS-PLGA NPs). The encapsulation efficiency (%EE) of bound mRNA:LPNs and mRNA:CS-PLGA NPs was evaluated indirectly by pelleting all samples down at 24,400*g* for 30 min and determining the concentration of unbound mRNA in the supernatant by measuring absorbance at 260/280 nm with a NanoDrop Spectrophotometer. This enabled the calculation of bound mRNA multiplied by a factor of 100 to receive the percentage encapsulation efficiency. Four independent batches of mRNA-loaded NPs were produced and characterized to obtain the hydrodynamic size, PDI and ζ-potential while the morphology assessed with conventional SEM and TEM after staining with 0.5% w/v PTA solution.

### Determination of mRNA Binding and release by gel retardation assay

The determination of mRNA binding and its stability within the nanoparticles were analyzed by a gel retardation assay using 0.75% (w/v) agarose gel electrophoresis and tested for all mRNA complexed NPs with varying weight ratios (1:10, 1:20 and 1:30). To further induce a release of complexed mRNA, 5 μL heparin (30 mg/mL; Sigma-Aldrich, Darmstadt, Germany) was added to the mRNA complexed NPs and incubated for 15 min at room temperature. All samples were then mixed with 2 μL of orange DNA loading dye (6×, Thermo Fisher Scientific, Waltham, MA, USA), loaded into the agarose (Serva, Heidelberg, Germany) gel containing 3 μL of ethidium bromide (10 mg/mL; Sigma-Aldrich, Darmstadt, Germany) and run for 25 min at 90 V using 0.5× TBE buffer. The mRNA bands were then visualized with a UV illuminator (Fusion FX7 imaging system from Peqlab, Erlangen, Germany).

### Cell cultures

Bone-marrow-derived murine dendritic cell line (DC2.4 cell, Cat# SCC142, Merck Millipore, Darmstadt, Germany) was maintained in complete media comprised of DMEM supplemented with 10% FCS, 10 mM HEPES, 2 mM l-glutamine and cultured in a humidified incubator at 37 °C with 5% CO_2_. A human lung carcinoma cell line (A549 cells; NO. ACC 107, DSMZ GmbH, Braunschweig, Germany) was grown in complete cell culture medium (RPMI 1640; PAA laboratories GmbH, Pasching, Austria) with 10% FCS and used as model cell line to test the transfection efficiency of mRNA complexed NPs. Both cell lines were passaged with trypsin–EDTA upon reaching ~ 85% confluency and used for the experimental settings as described in the next sections.

### Cell viability and cytotoxicity assay: live-dead staining

DC2.4 cells were seeded in a 24 well plate at a density of 3 × 10^5^ cells/well, in 500 μL of DMEM with 10% FCS and incubated overnight at 37 °C with 5% CO_2_ to allow complete adherence of cells to the well. The next day, cell culture medium was removed, and cells were washed twice with HBSS. Prior to the experiment, blank LPNs and CS-PLGA NPs were prepared at appropriate concentrations (10, 20, 40, 60, 100 and 160 μg/mL) in HBSS and cells were incubated with 500 μL of each concentration for 4 h under slightly shaking at 37 °C with 5% CO_2_. Cells treated with HBSS buffer only were used as negative control. For the positive control (dead cells), cells were plated in a separate 24 well plate and incubated for 1 h at 56 °C. Immediately after incubation, cells were washed twice with PBS, detached with trypsin–EDTA and placed in round-bottom falcon tubes (Falcon, A Corning Brand, Tamaulipas, Mexico). A volume of 200 μL of FACS-buffer (containing 2% FCS in PBS) and 1 mL of PBS were added to cells and centrifuged at 4 °C and 300*g* for 5 min. The supernatant was discarded, and the pellets were washed once again with 1 mL PBS and centrifuged respectively. A Live/Dead Fixable Staining Kit 568/583 (PromoKine, Promocell, Heidelberg, Germany)—suitable for flow cytometry—was used to quantify cell viability and cytotoxicity. The dye within the kit was reconstituted, and cells were further treated according to manufacturer’s protocol. Thus, the remaining pellet after the second centrifugation was re-suspended in 1 mL PBS, and thereafter, 1 μL of fixable dead cell stain was added to the cells, mixed well and further incubated protected from light at 4 °C for 30 min. Cells were then washed with 1 mL PBS, fixed in 1% paraformaldehyde (Electron Microscopy, PA, USA) and stored until analysis. Cell viability and cytotoxicity of tested NPs was quantified using flow cytometry (BD LSRFortessa™ Cell Analyzer, Biosciences, Heidelberg, Germany) in the PE-channel. Fifty thousand cells per sample were measured by the cytometer and obtained data were analyzed using FlowJo software (FlowJo 7.6.5, FlowJo LLC, OR, USA). Three independent experiments were conducted, and results are expressed as the mean ± SD.

### Cellular uptake in DC2.4 cell line

To quantify the cellular localization of blank and mRNA complexed LPNs and CS-PLGA NPs, a green fluorescent dye (fluoresceinamine, FA with a peak emission wavelength of 530 nm) was first covalently coupled to PLGA before nanoparticle-production. DC2.4 cells were seeded and grown as described for the live-dead staining assay. Prior to the experiments, fluorescent NP types, with and without mRNA (FA-LPNs, mRNA:FA-LPNS, CS-FA-PLGA and mRNA:CS-FA_PLGA NPs) were prepared in HBSS buffer at different concentrations (20, 40 and 60 μg/mL). A volume of 500 μL of each NP suspension was then applied to the cells at 37 °C and 5% CO_2_ 2 h or 4 h on a shake at 150 rpm.

Right after each time-point, cells were rinsed twice with HBSS buffer in order remove excess of NPs, detached from the well with trypsin, placed in round-bottom tubes, and centrifuged down to receive the cell pellets. Cells were then re-suspended and fixed for flow cytometry. Fifty thousand cells were assessed using the FITC fluorescence channel. The percentage of cells positive to FA-labeled NPs were determined with FlowJo. Results are presented as the mean ± SD of four independent experiments.

### In vitro transfection and kinetics

To test whether mRNA complexed NPs would efficiently transfect dendritic cells, DC2.4 cells were seeded in 24 well plates at a density of 3 × 10^5^ cells per well in 500 μL DMEM cell culture medium with 10% FCS. Non-labeled LPNs and CS-PLGA NPs containing 1 μg of mRNA were prepared with a mRNA:NPs weight ratio of 1:10, 1:20 and 1:30 in 500 μL HBSS buffer. The cells were then washed twice with HBSS buffer and incubated with the mRNA complexed NPs for 2 h and 4 h. In an modified experimental setting, cells were exposed to NPs for 4 h, NPS were removed by washing, and cells were then incubate with NP-free DMEM plus 10% FCS for 24 h and 48 h. The different mRNA:NPs weight ratios and time-points were used to identify the maximum transgene expression. A commercially available transfection reagent, JetPRIME^®^, was used as positive control, whereas cells treated with naked mRNA-mCherry and medium alone were used as negative control. The efficiency of mCherry transgene expression was quantified using flow cytometry, and cytometer settings were adjusted to differentiate between transfected and non-transfected cells. Data were analyzed with FlowJo and results are shown as mean ± SD of four independent experiments.

Furthermore, DC2.4 cells incubated for 24 h and 48 h post-transfection were visualized by confocal laser scanning microscope (CLSM; Leica TCS SP 8, Leica, Wetzlar, Germany). Here, cell membranes were stained with Wheat Germ Agglutinin linked to Alexa Fluor 488 fluorescent dye (WGA, 10 μg/mL) and cell nuclei with 4′,6-diamidino-2-phenylindole (DAPI, 0.1 μg/mL). Samples were then fixed with 3% paraformaldehyde and stored at 4 °C until imaging. The images were acquired with a 25× water immersion objective at 1024 × 1024 resolution and afterwards processed with LAS X software (LAS X 1.8.013370, Leica Microsystems, Leica, Germany). CLSM visualization of transgene expression was repeated in three independent experiments and images were processed using the software ImageJ (Fiji).

### Live cell video microscopy: recording NP uptake and protein translation

To analyze the transgene expression kinetics of single cells over a time-course of 4 h, live cell imaging was performed in DC2.4 cells using fluorescently-labeled and mRNA complexed mRNA:FA-LPNs with a weight ratio of 1:30. NP uptake could be tracked due to the green fluorescent signal of FA-PLGA, while transgene expression of cells could be determined by following the red fluorescence emitted by the peptide translated from mCherry-mRNA. Here, 3 × 10^5^ cells/well were seeded overnight in a μ-Slide 8 well glass bottom chamber (ibidi, Martinsried, Germany) in DMEM with 10% FCS. CLSM facility was set to maintain constant physiological conditions for cells. The entire microscope was enclosed in a humidified incubation chamber enabling temperature regulation to 37 °C. The specimen was placed under a perfusion chamber to maintain a CO_2_ atmosphere at 5% with a flow speed of 3 L/h. Before recording, cell culture medium was replaced by HBSS and DC2.4 cells were placed under the microscope. Images were acquired using a 25× water immersion objective at 1024 × 1024 resolution with an interval of 3 min/image for a time frame of 4 h. Image acquisition was started Immediately after applying mRNA:FA-LPNs. Images were processed, compiled with the LAS X software and four different videos were produced with at 6 frames/s. NP uptake and the time-dependent change of transgene expression within single cells were analyzed by plotting the red (transgene expression) and green (FA-PLGA of NPs) fluorescence signals against time. To extract arbitrary fluorescence values from the microscopy images, we used a science-distribution tool of the software ImageJ. Each video was loaded and split in its three channels (bright field microscopy, red fluorescence and green fluorescence). All videos were captured at four focal planes, which were merged with Z Project, while keeping the maximum intensity across the planes for each pixel. The videos were converted to 8-bit grayscale and saved as Tagged Image File format (TIF). To analyze the transgene expression kinetics, the aforementioned channels of each video were opened in the 80th frame corresponding to the 4 h time-point after the start of the experiment. In this particular frame, successfully transfected cells could be easily identified in the red channel. For each video, we located 8 red-fluorescent (transfected) and 2 non-fluorescent cells (non-transfected) in the 80th frame, matching the 8:2 transfection ratio observed by flow cytometry. With the selection tool of *Fiji,* the boundaries of these 10 cells were selected in the bright filed channel and saved as regions of interest (ROI). These ROIs were subsequently used in the green and red fluorescence channel to determine the fluorescence intensity in several frames. The same analysis was repeated for frames 70, 60, 50, 40, 30, 20, 10 and 1 (matching with the time-points of: 3.5 h, 3 h, 2.5 h, 2 h, 1.5 h, 1 h, 0.5 h and 0 h). Finally, the time-dependent change of the signal-intensity was plotted using the mean value of fluorescence intensity.

### Potential of LPNs and CS-PLGA NPs for functional mRNA-delivery in A549 cells

A549 cells were used to show the potential of both NPs to efficiently transfect epithelial cells. A549 cells were seeded at a density of 1 × 10^5^ cells/well in 24 well plates. The experimental setting with further quantification and visualization of transgene expression in A549 cells was performed as described before for DC2.4 cell. The used mRNA complexed non-labeled NPs were incubated for 4 h and transfected cells were counted 24 h and 48 h post-transfection with flow cytometry and further visualized using CLSM.

### Statistical analysis

Statistical analysis was performed using two-way ANOVA followed by a Tukey’s multiple comparison test with the software Graph Pad Prism 6 for Windows (Version 6.01, GraphPad Software Inc.). Data were considered as statistical significant for p < 0.05 (*p < 0.05, **p < 0.01, ***p < 0.001 and ****p < 0.0001). N is the number of independent experiments and n is the number of technical replicates.

## Results and discussion

### Design and characterization of blank lipid-coated and chitosan-coated PLGA nanoparticles

Two different core–shell structured delivery systems have been manufactured using either PLGA, a biodegradable and biocompatible polymer as the core, which was coated with a cationic lipid (DOTMA), or a cationic polymer (chitosan), to achieve a positive surface charge. In either case, we produced the blank (i.e. mRNA-free) particles first and complexed them with mRNA in a second step before application. The production procedure and all related settings for both NPs were kept equal to enable a good foundation for comparison. In the first experimental setting, we designed blank lipid-coated PLGA nanoparticles (LPNs) as described previously [17, 27] and chitosan-coated PLGA Nanoparticles (CS-PLGA NPs) [10, 28] using a modified double-emulsion method with PVA as stabilizer and analyzed their physicochemical properties in regards to hydrodynamic size, polydispersity index (PDI) and ζ-potential. The size as well as surface charge of nanoparticles are likely to influence pharmacokinetics, accumulation behavior, tissue distribution, cellular internalization [20, 32] and their cytotoxic effect [14]. Therefore, these two parameters were diligently taken in consideration. Particle sizes were kept below 250 nm to qualify endocytosis, and the surface potential not to exceed + 25 mV as strong cationic surface charge correlates with higher cytotoxic effects [14]. The mean hydrodynamic size of LPNs was 230 nm, with a narrow PDI of approximately 0.09 revealing a cationic surface potential of around + 16 mV respectively (Table 1). In comparison to the lipid–polymer NPs, CS-PLGA NPs exhibit a smaller particle size of about 140 nm (PDI: 0.12) with an equal surface charge in the positive range of + T25 mV (Table 1).

**Table 1:** Physicochemical characteristics of blank and mRNA complexed nanoparticles (NPs) with the percentage encapsulation efficiency (%EE) of mRNA

Nanoparticles	Size (nm)	PDI	ζ-potential (mV)	%EE
Blank LPNs	231 ± 7.0	0.09 ± 0.02	16.25 ± 3.24	–
*mRNA:LPNs_1:10	322.2 ± 26.1	0.21 ± 0.02	− 0.05 ± 6.54	91.3 ± 1.5
*mRNA:LPNs_1:20	217.5 ± 11.6	0.16 ± 0.04	19.62 ± 1.13	87.0 ± 2.0
*mRNA:LPNs_1:30	*243.5 ± 45.3*	*0.18 ± 0.08*	*22.97 ± 0.68*	*92.8 ± 2.0*
Blank CS-PLGA NPs	141.2 ± 6.2	0.12 ± 0.01	25.20 ± 3.84	–
*mRNA:CS-PLGA_1:10	176.4 ± 5.5	0.32 ± 0.00	13.30 ± 2.76	87.0 ± 0.4
*mRNA:CS-PLGA_1:20	158.6 ± 1.7	0.23 ± 0.00	20.42 ± 1.22	95.4 ± 0.6
*mRNA:CS-PLGA_1:30	*151.3 ± 3.4*	*0.18 ± 0.02*	*25.55 ± 1.22*	*94.2 ± 0.3*

Samples highlighted in italics indicate the NPs with best colloidal properties. *mRNA:NPs weight ratio. *N* = 4, mean ± SD

Keeping in mind the potential biopharmaceutical application, we investigated the storage stability of LPNs at 4 °C and room temperature (RT) post-preparation for a time-period of 62 days by measuring the particle size, PDI, and ζ-potential, respectively, which revealed no significant difference for all tested conditions (Additional file 1: Figure S1).

The stability and hence aggregation behavior of the particles under different physiological conditions was further investigated following 2 h, 4 h and 24 h incubation time at 37 °C, which is essential to understand the behavior of nanoparticles in a biologically relevant environment. LPNs and CS-PLGA NPs were incubated in HBSS buffer, DMEM, and DMEM plus 10% FCS and their colloidal stability was measured after the predetermined time-points. LPNs remained overall stable in all tested media. A significant decrease in the hydrodynamic size from approx. 220 nm to approx. 150 nm with an increase in PDI to approx. 0.35 was observed in serum containing medium only (Additional file 1: Figure S2A1, A2). By contrast, CS-PLGA NPs show a significant increase in size and PDI after exposure to all tested media compared to the untreated control (Additional file 1: Figure S2B1, B2) while the addition of serum to DMEM resulted in a decreased size with a substantial strong increase of PDI from approx. 0.1 up to around 0.5. The significant change in colloidal characteristics after addition of serum, especially the decrease in size was already reported in Schulze et al. [33]. This effect is evoked by adsorption of negatively charged serum proteins onto the surface of the positively charged nanoparticles, causing destabilization of the particles. However, for both NP types the hydrodynamic size showed some changes upon transfer in buffer or medium on the short time scale but then remained stable for 24 h.

### Structural visualization of blank nanoparticles

**Fig. 2:**
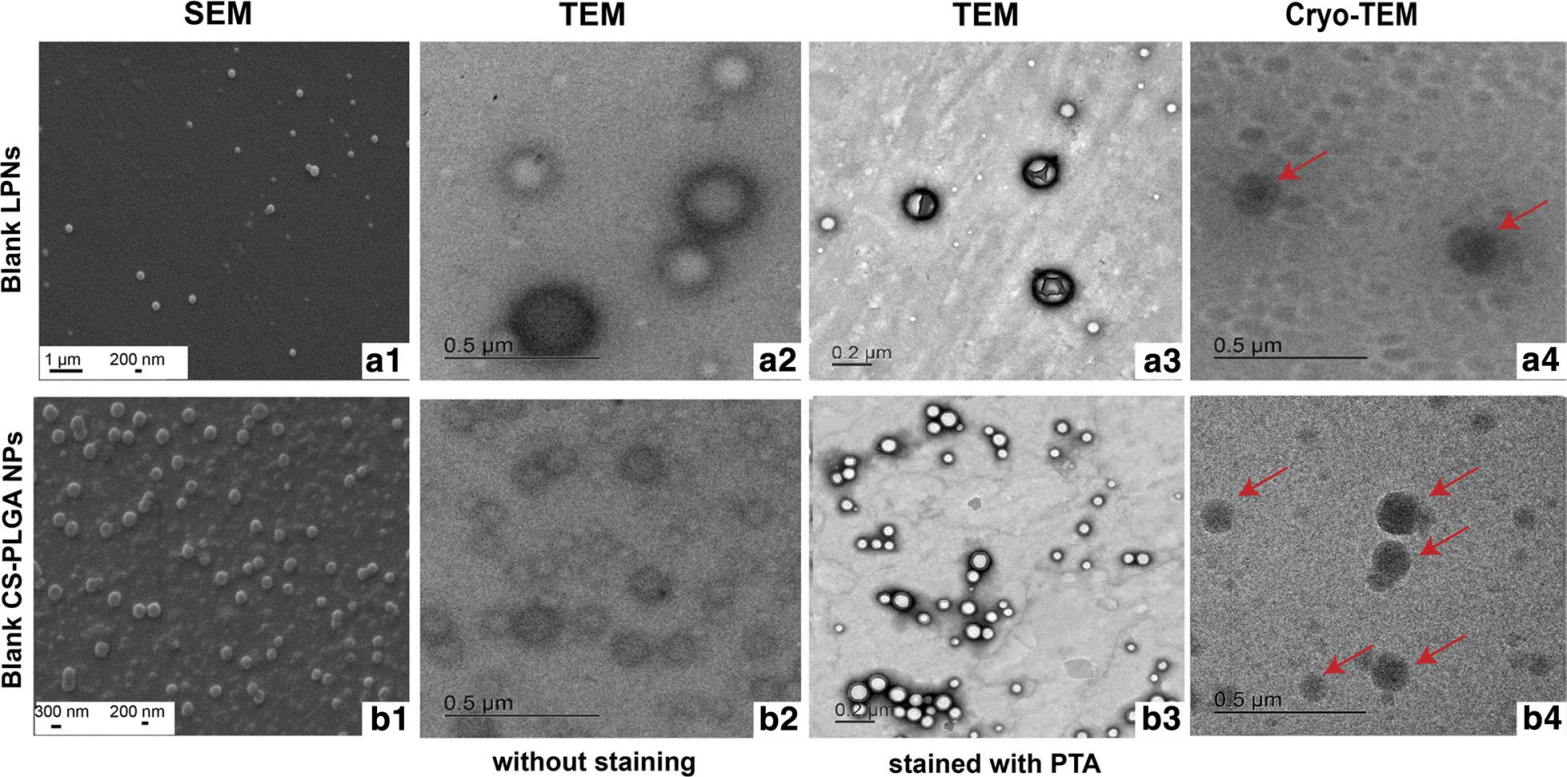
A variety of different microscopic methods used to visualize blank LPNs (**a1**–**a4**) and CS-PLGA NPs (**b1**–**b4**). **a1**, **b1** SEM images show a smooth, spherical morphology of the nanoparticles, **a2**, **b2** TEM images of particles without staining give a hint about the core–shell structure, seen in a dark electron dense layer surrounding a brighter core. **a3**, **b3** To emphasis this structure and increase the contrast, the particles were stained with 0.5% wt/V PTA and imaged with TEM and further unstained nanoparticles were visualized with a less harsh method using Cryo-TEM with red arrows pointing to the particles (**a4**, **b4**). Scanning electron microscopy (SEM), transmission electron microscopy (TEM)

Besides the criteria of well-defined size and ζ-potential, the shape and structure of nanoparticles are further important parameters impacting cellular internalization [34]. Hence, we have employed a variety of techniques using conventional imaging by scanning electron microscopy (SEM), transmission electron microscopy (TEM) and cryo-TEM to visualize NP morphology and structure, as all of the techniques have their specific advantages and limitations. We further improved the TEM-images by staining the nanoparticles with PTA. As SEM produces images of the sample by scanning the surface with an electron beam, blank LPNs and CS-PLGA NPs revealed a smooth and evenly shaped spherical surface structure (Fig. 2a1, b1). We then analyzed the inner structure of the nanoparticles by using TEM (Fig. 2a2, b2) in order to characterize the arrangement of used excipients as it was discussed in literature that lipid–polymer nanoparticles have a core–shell like structure [35]. Here, the images reveal a darker and hence electron dense layer surrounding a brighter core. The anionic PTA stain showed strong adherence to the cationic particles surface without entering the particle core, which may further emphasize a core–shell structure of both NPs (Fig. 2a3, b3). Furthermore, Cryo-TEM was implemented as a technique with minimal risk of artifact formation [36] (e.g. agglomeration, particle size shrinkage) and confirmed the spherical structure of the NPs (Fig. 2a4, b4). The size of both NPs found by Cryo-TEM was in the range of 50–200 nm.

### Complexation and characterization of mRNA-loaded LPNs and CS-PLGA NPs

The cationic head group of the lipid and the cationic amine group of chitosan facilitates the electrostatic interaction and hence binding of the net anionic charged mRNA to the surface of the nanoparticles.

The NPs have different ζ-potential depending on the cationic coating and the usage of fluorescence labeled PLGA. Thus, same weight ratios of mRNA loading lead to different charge neutralization and destabilization. The ratio of mRNA:NP affects the colloidal stability since the nucleotides neutralize the cationic particle charge either partly, fully or reverse it to anionic, implicating a risk of particle-aggregation when the ζ-potential approaches zero. For reasons of better comparability, mRNA:NPs weight ratios (w/w) of 1:10, 1:20 and 1:30 were arbitrary selected. A model mRNA encoding for the red fluorescent reporter gene mCherry was used and hence complexed onto the surface of LPNs and CS-PLGA NPs. This design has the following advantages (i) to avoid exposure of mRNA to the harsh conditions of NP production (e.g. high shear stress), (ii) to protect mRNA after complexation against nucleases and (iii) for fast release upon certain physiological triggers (e.g. competing endogenous anions) as necessary for effective translation.

**Fig. 3:**
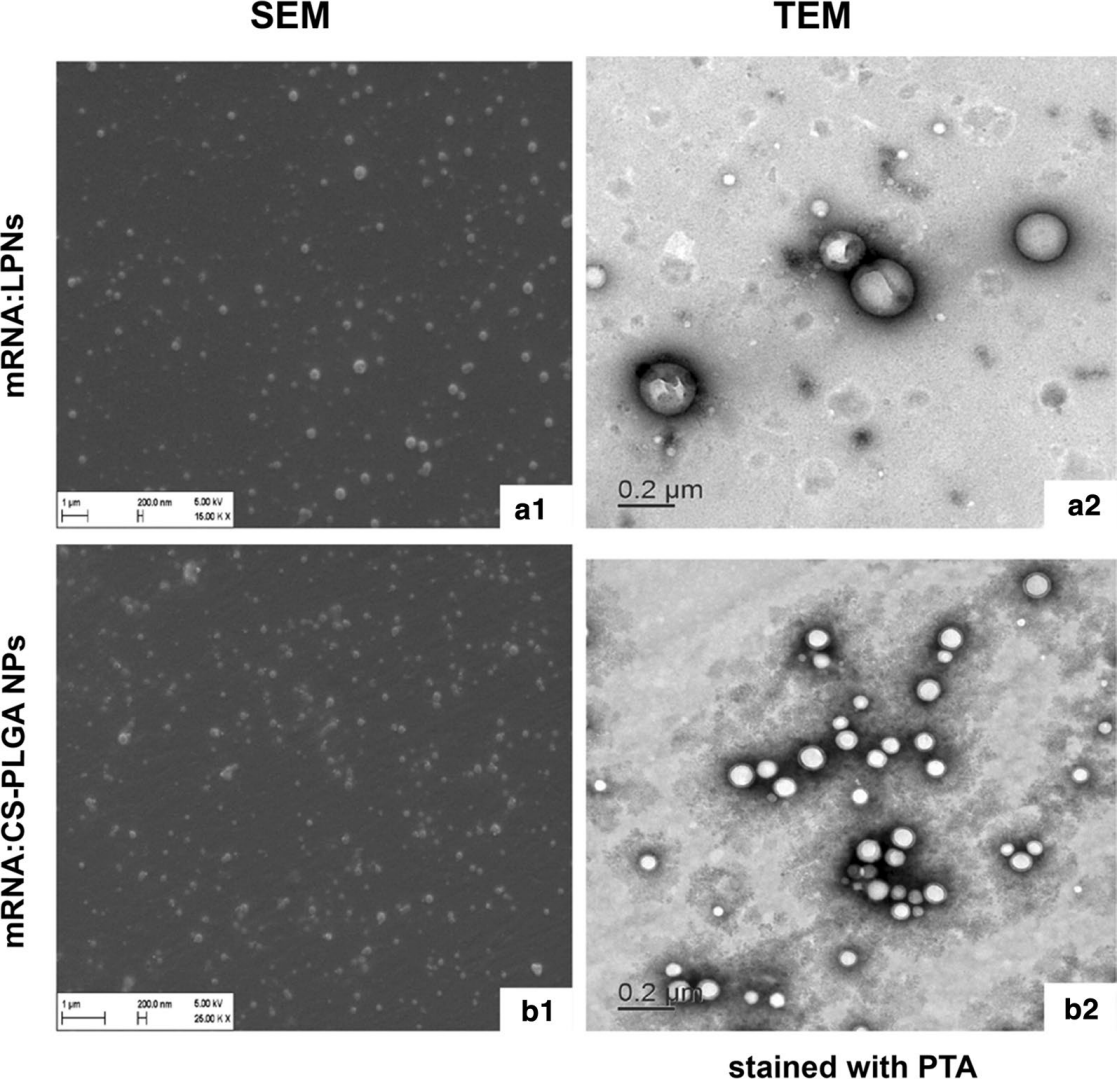
Morphology of mRNA-loaded LPNs (**a1**, **a2**) and CS-PLGA NPs (**b1**, **b2**) visualized using SEM and TEM. **a1**, **b1** SEM images show a smooth, spherical morphology of the mRNA-loaded nanoparticles and **a2**, **b2** TEM images show the core–shell structure of the particles after staining with 0.5% (w/V) PTA

**Fig. 4:**
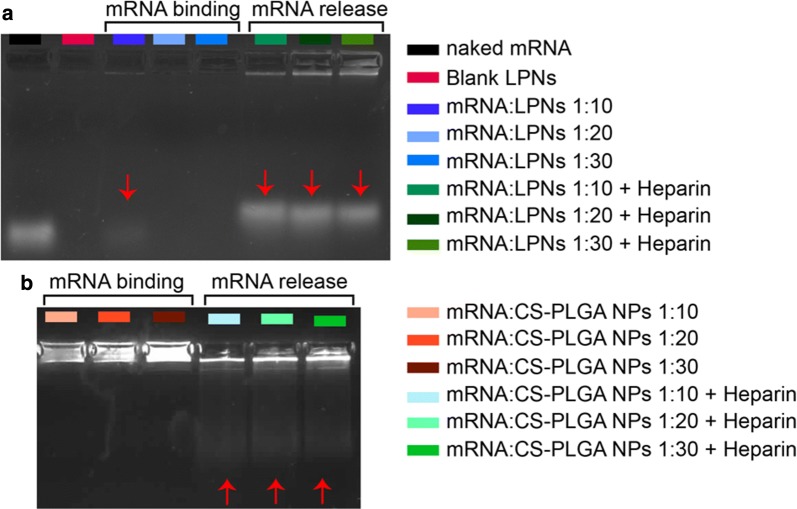
Gel retardation assay of mRNA complexed with different nanoparticles (NPs) at various ratios: **a** LPNs and **b** CS-PLGA NPs. Both images indicate mRNA binding to NPs and their appropriate release using heparin

The physicochemical properties in terms of hydrodynamic size, PDI, ζ-potential were characterized and the encapsulation efficiency quantified respectively (Table 1). mRNA-loaded LNPs (mRNA:LNPs) at a ratio of 1:10 show an increment of the size from ~ 230 nm (blank LPNs) to ~ 322 nm, following a broader size distribution profile (highest PDI value of 0.21) and a strong decrease in the ζ-potential from ~ 16 mV to nearly 0 mV (Table 1) indicating a destabilized system. Besides, the studied weight ratios of 1:20 and 1:30 resemble in their colloidal properties regarding size and ζ-potential, while the PDI shows a slight increase. The same tendency upon various tested weight ratios was observable for mRNA-loaded CS-PLGA NPs (mRNA:CS-PLGA NPs). Both delivery systems complexed the mRNA up to ~ 90% (Table 1), maintaining spherical morphology (Fig. 3a1, a2, b1 and b2). The increment of NPs concentration correlates with higher amount of amine groups of the used cationic excipients, enabling a better saturation and hence stronger and improved condensation of the anionic mRNA. Additionally, agarose gel electrophoresis elucidated the efficiency of both NPs to bind mRNA-mCherry (Fig. 4). For mRNA-loaded LPNs of 1:10 w/w, a band of free mRNA was observable in the gel in contrast to 1:20 and 1:30, indicating stronger condensation of mRNA with increasing amount of NPs (Fig. 4a).

Surprisingly, a fluorescence signal of EtBr in the bags for all ratios of mRNA:LPNs, as an indicator for the presence of mRNA with the nanoparticles, was not seen (Fig. 4a, mRNA binding). This is presumably attributed to the used lipid DOTMA interacting stronger with the mRNA and hence impeding the further intercalation of EtBr. The presence of mRNA-mCherry associated onto the surface of LPNs was, however, demonstrated using heparin, which causes a fast release of mRNA within 15 min as seen through the free bands in the gel (Fig. 4a, mRNA release). In comparison, mRNA:CS-PLGA NPs allow intercalation of EtBr as seen in Fig. 4b, as no band could run through the gel, while the addition of heparin just partially released the mRNA from CS-PLGA NPs (Fig. 4b, mRNA release). Furthermore, the visible bands at the bottom of agarose gel pockets of Fig. 4 can be explained by an incomplete release of mRNA from the NPs after heparin treatment, preventing the motion of particle-bound mRNA through the gel. The mRNA migrating deeper into the gel is only a fraction of a total amount of mRNA bound to the particle. This effect is even more pronounced in the chitosan sample than the LNP sample.

### Cell viability and cytotoxicity assay

**Fig. 5:**
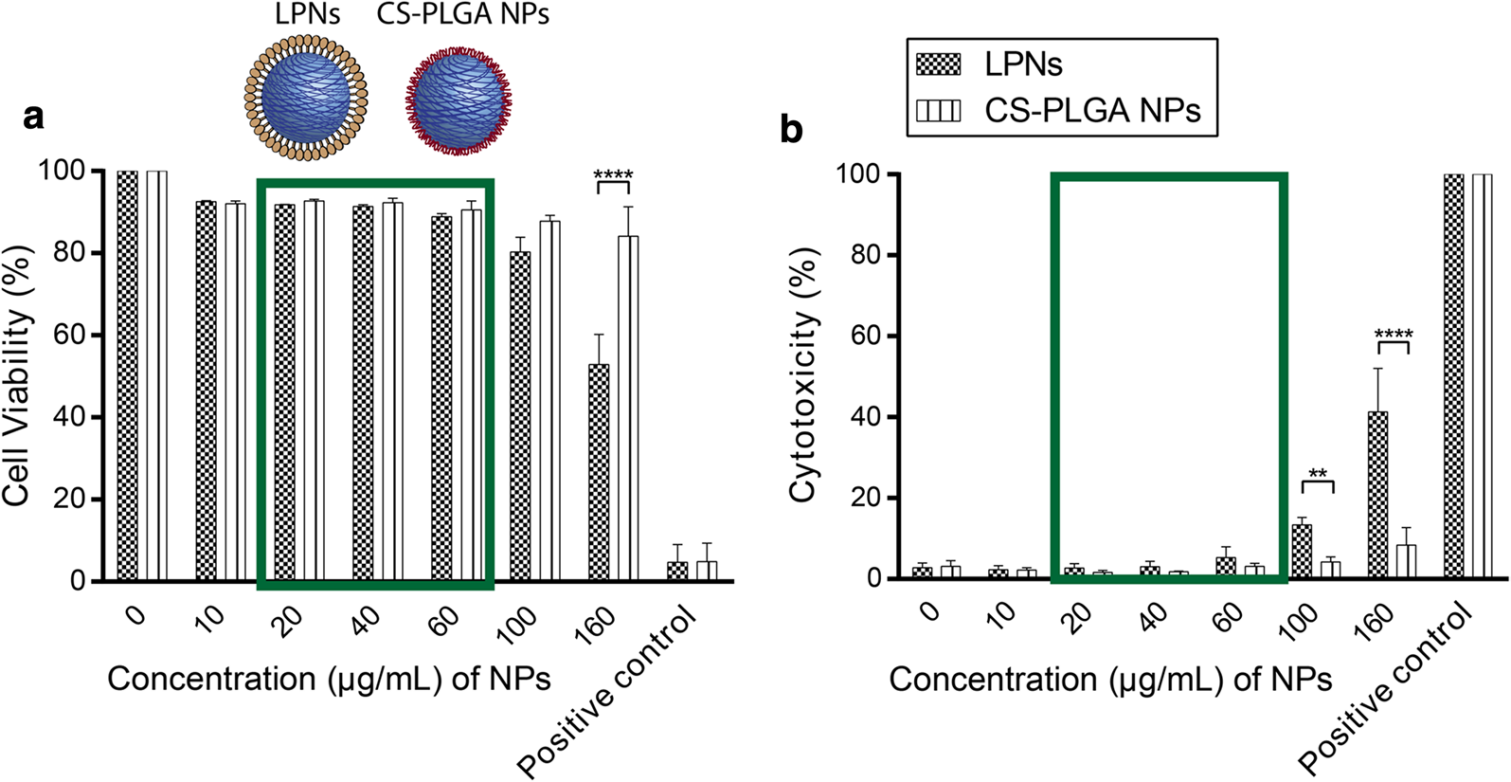
**a** Cell viability and **b** cytotoxicity studies assayed with a live-dead staining kit on DC2.4 cells using blank LPNs and CS-PLGA NPs. Particles were incubated for 4 h: LPNs reveal a slightly higher cytotoxicity than CS-PLGA NPs. Green rectangles represent the concentration range used for cellular localization and transfection studies. *N* = 3, mean ± SD (**p < 0.01, ****p < 0.0001)

It is common knowledge that cationic charged particles are associated with higher cytotoxic effects [37], partly explicable due to potentially enhanced interaction of cationic particles with the anionic charged cell membrane, which however also facilitate cellular uptake [38]. Therefore, we have analyzed the cell viability of blank LPNs and CS-PLGA NPs on DC2.4 cells, by nanoparticle incubation at different concentrations for 4 h (Fig. 5a, b) and differentiated dead from living cells by staining with a live-dead staining kit with an appropriate gating strategy (Additional file 1: Figure S3). Under such conditions, lipid-PLGA nanoparticles showed no cytotoxic effects up to a concentration of 100 µg/mL, which were only observed at 160 µg/mL, as reflected by a drop of cell viability to ~ 50%. CS-PLGA NPs demonstrated no toxic effects over the tested concentration ranges. In order to keep a well-tolerated concentration range for our further cell studies, we used the ranges marked with green rectangles (Fig. 5a, b). The toxicity difference at a higher concentration between lipid-PLGA and chitosan-PLGA nanoparticles might be associated with the pH sensitivity of the primary ammonium group in chitosan showing changing deprotonation degree depending on the surrounding pH value in comparison to the quaternary ammonium group in DOTMA. The HBSS-buffer used for the toxicity studies has a pH of 7.4 a value in which chitosan reveals a slight diminishing protonation degree resulting in a decrease of ζ-potential and presumably less cellular interaction [31]. Additionally to that, the hydrophobic nature of the DOTMA envelope might elicit a better interaction with cells and hence uptake.

### Kinetics of cellular internalization for blank and mRNA-loaded nanoparticles

**Fig. 6:**
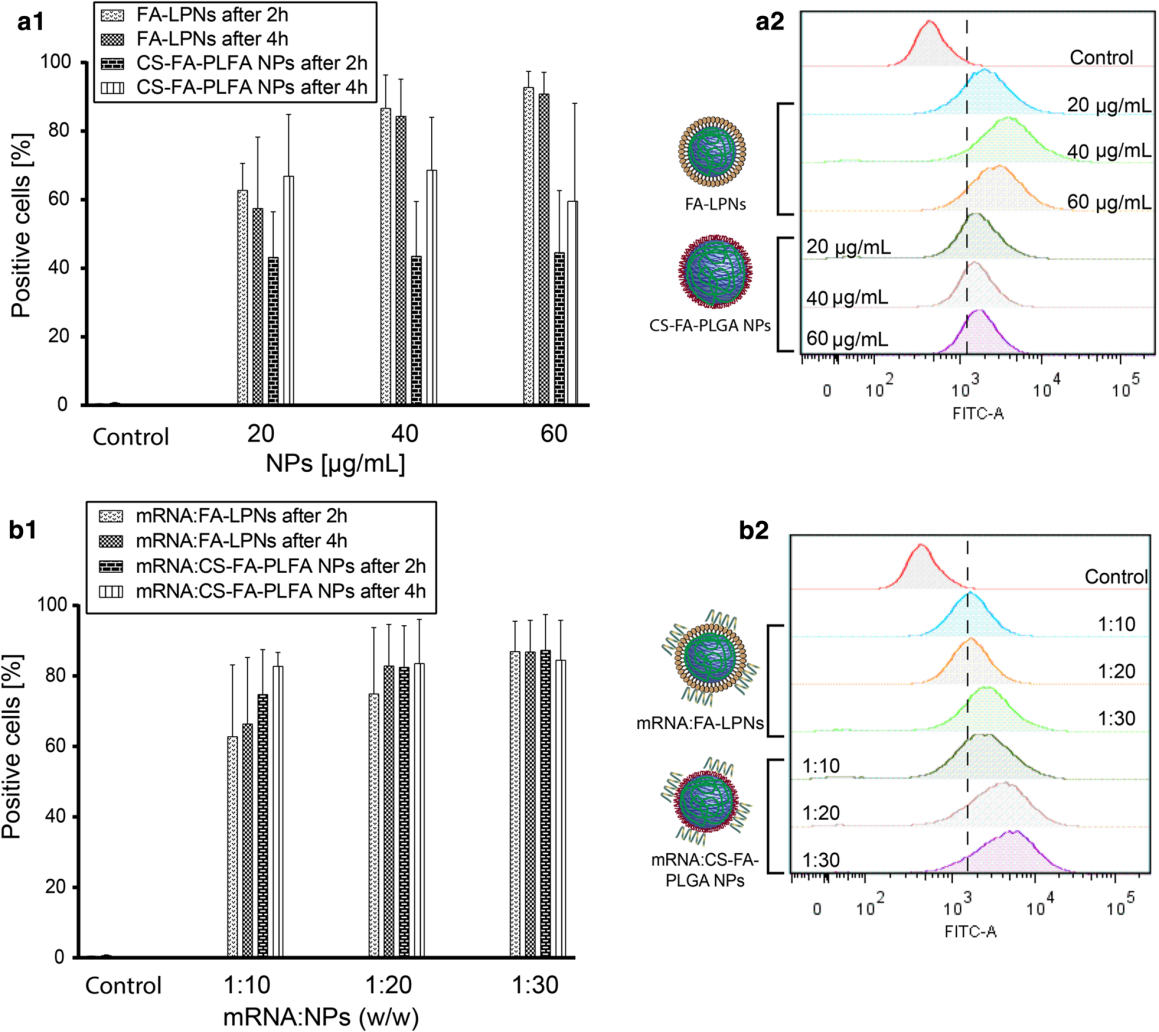
Quantification of cellular NP association for fluoresceinamine (FA) labeled blank (**a1**, **a2**) and mRNA complexed nanoparticles (**b1**, **b2**) tested in DC2.4 cells. NPs were incubated for 2 h and 4 h at different concentrations (20, 40 and 60 µg/mL corresponding to the weight ratios 1:10, 1:20 and 1:30 used for transfection). **a1**, **b1** Green NP fluorescent signal quantified by flow cytometry. Blank LPNs tend to show more uptake then blank CS-PLGA NPs, whereas for mRNA-loaded nanoparticles the opposite was observed. None of the samples showed a significant difference between 2 and 4 h incubation. **a2**, **b2** Representative graphs obtained for NP samples after 4 h incubation. *N* = 4, mean ± SD

Prior to transfection studies, we evaluated the kinetics of cellular internalization for blank and mRNA-loaded NPs, as the knowledge about the efficiency of uptake towards dendritic cells might help to understand the subsequent transfection. To quantify the internalization of nanoparticles into cells, we used fluorescently labeled particles, by first covalently coupling fluoresceinamine to PLGA and subsequently designing and characterizing appropriate nanoparticles. Labeled blank LPNs (FA-LPNs) show similar colloidal characteristics as non-labeled LPNs, while labeled blank CS-PLGA NPs (CS-FA-PLGA NPs) indicated a drop of ζ-potential from approx. + 25 mV (for non-labeled) to + 10 mV (Additional file 1: Figure S4). The attachment of chitosan to the PLGA core is mainly driven by charge interaction. As the conjugation of FA to the carboxyl group decreases the anionic charge of the PLGA core, less chitosan can bind to PLGA causing a decrease in ζ-potential. In comparison, DOTMA can additionally attach to the PLGA core by hydrophobic interaction and might be less dependent on the anionic nature of the PLGA core. mRNA-loaded labeled NPs (either mRNA:FA-LPNs or mRNA:CS-FA-PLGA NPs) were prepared at three different mRNA:NPs ratios 1:10, 1:20 and 1:30 comparable to a particle concentration of 20 µg/mL, 40 µg/mL and 60 µg/mL, resp., and characterized for their physicochemical properties. While the size and PDI for both mRNA-loaded labeled NPs at different ratios show no significant difference, the ζ-potential indicates a strong decrease to a negative range for the tested ratios, only mRNA:LPNs 1:30 remaining in the positive range (Additional file 1: Figure S4). The ζ-potential of the FA-labeled NPs was lower than the non-labeled NP of the same type. Thus, lower mRNA ratios already neutralize the particle charge. The effect of NPs internalization by dendritic cells was then analyzed at the same three concentrations for labeled blank and mRNA-loaded NPs and at two different time-points, i.e. after 2 h and 4 h exposure to cells at 37 °C. Right after NPs exposure, cells were trypsinized and analyzed by flow cytometry. We could first observe a higher cell association for FA-LPNs over CS-FA-PLGA NPs (Fig. 5a1). FA-LPNs showed a cell association with nearly 95% of the cells for concentrations ≥ 40 µg/mL and hence a strong fluorescence shift (Fig. 6a1, a2). CS-FA-PLGA NPs revealed no concentration-dependent cellular internalization and lower percentage (approx. 70%) of cells with particle association. Although CS-FA-PLGA NPs show some increase NP positive cells after 4 h, no clear time-dependent cell association of the particles was observable.

When either type of labeled NPs was complexed with mRNA and exposed to DCs, the cell association behavior changed in comparison to the labeled blank particles. All mRNA-loaded particles indicate no significant concentration or time-dependent association, but enhanced cellular association as seen in the higher amount of particle-positive cells (Fig. 6b1, b2). Hence, for all tested nanoparticles the surface charge does not play a role in the efficiency of cell association, as mRNA-loaded FA-labeled nanoparticles show a negative surface charge in comparison the blank ones. Another point needed to be taken in consideration is the type of cells used in this study. DC2.4 cells belong to phagocytic and professional antigen presenting cells. Once a foreign material is recognized by immature DCs it will be endocytosed, DCs mature with changed metabolism and downregulated phagocytosis [39]. Thus, in their immature state, they have a higher phagocytic and thereby higher uptake activity compared with non-phagocytic cells [38] and hence show a similar uptake behavior towards negatively and positively charged nanoparticles. After reaching a saturation state following a downregulated metabolism, the phagocytic activity decreases and NPs are no further taken up, which explains the concentration- and time-independence of the NPs internalization. We further assume that the adsorption of mRNA improves the cell association due to better stabilization of the nanoparticles after mRNA adsorption onto the surface. Sue et al. did observe similar effect for a comparable system [11]. This behavior is clearly seen in the higher cell internalization of mRNA:CS-FA-PLGA NPs compared with the blank particles. The high amount of positive cells upon incubation with FA-LPNs compared to CS-FA-PLGA is assumedly a result of the hydrophobic nature of FA-LPNs.

### Kinetics of protein translation in dendritic cells

**Fig. 7:**
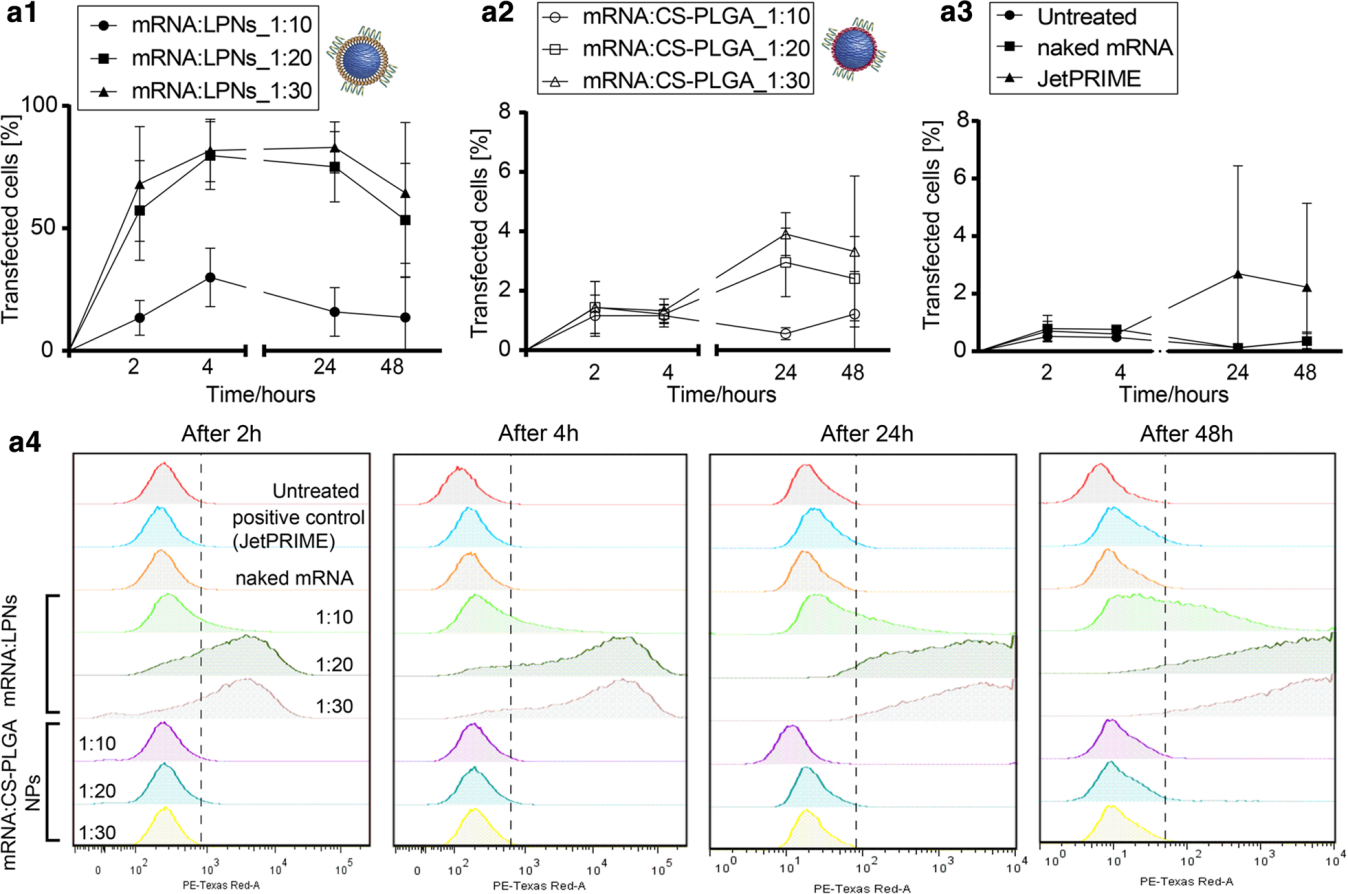
The kinetics of transfection for both mRNA-loaded NPs was quantified using flow cytometry, which indicated a significant difference between **a1** mRNA:LPNs over **a2** mRNA:CS-PLGA NPs, while mRNA:LPNs with a ratio of 1:20 and 1:30 elucidated a significant higher transfection rate over 1:10. **a3** Control samples for transfection studies were JetPRIME^®^ as positive control and naked mRNA as negative control. (Note the enlarged y-axis, *N* = 4, mean ± SD.) **a4** Representative graphs for evaluated time-points demonstrate a strong fluorescence shift for mRNA:LPNs with an increment in time and hence a higher transgene expression

A variety of different non-viral systems has been introduced to deliver safe and efficiently mRNA to the site of action, especially for mRNA-therapeutics and mRNA-vaccination strategies. However, most of them still show limitation regarding their efficiency to transfect cells, as they have to cross several biological barriers while maintaining the functionality of the carried mRNA. As mRNA represents a transient cargo [6, 9] to be delivered into the cytoplasm, the kinetics of mRNA transgene expression was explored by taking measurements 2, 4 h after NPs exposure to DCs and additionally 24 and 48 h post-transfection. We further quantified and compared the efficiency of both mRNA-loaded LPNs and CS-PLGA NPs, with jetPRIME^®^ used as positive control. At a w/w ratio of 1:10, mRNA:LPNs revealed a transfection rate in DCs of around ~ 40%, which increased to nearly 80% at w/w ratios of 1:20 and 1:30 (Fig. 7a1). Correspondingly, a strong shift in the red fluorescence signal was seen in the dot-plots (Additional file 1: Figure S5). Already after 2 h, we could observe almost 20% transfected cells for ratio 1:10 following nearly 60% for the weight ratios of 1:20 and 1:30.

**Fig. 8:**
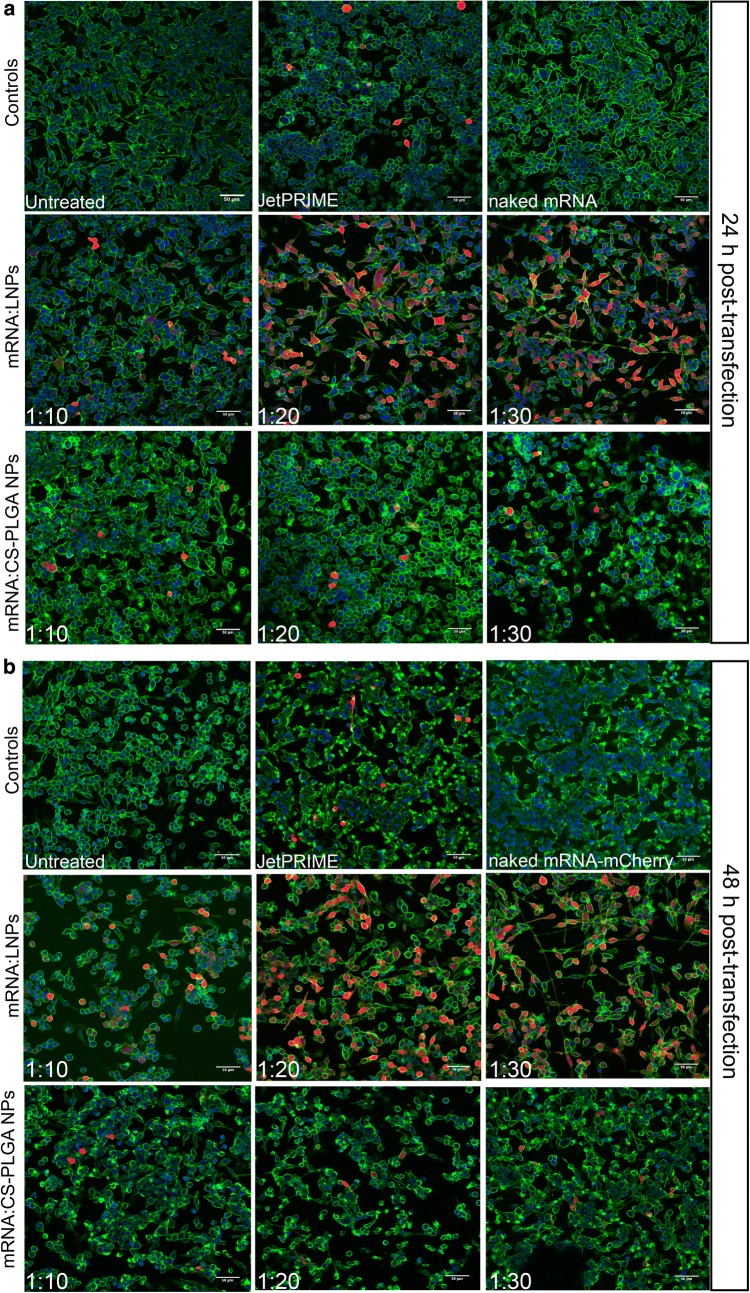
Representative confocal images of DC2.4 cells transfected with both mRNA complexed NPs and by using jetPRIME^®^ as a positive control, naked mRNA as negative control. Transfection was analyzed with CLSM **a** 24 h and **b** 48 h post-transfection. Red fluorescence reveals cells successfully transfected with the nanoparticles while their morphology remains consistent with non-transfected cells (staining: green: cell membrane; blue: cell nucleus; scale bar: 50 μm)

While mRNA:CS-PLGA NPs showed similar uptake behavior, protein translation rate was significantly less than for mRNA:LPNs, reaching only 5% of cells with a maximum after 24 h (Fig. 7a2). Translation rates for naked mRNA was almost same as the background of untreated cells and JetPRIME^®^ transfection efficacy was comparable to mRNA:CS-PLGA NP (Fig. 7a3). Furthermore, mRNA:LNPs elucidated the highest transfection efficiency already after 4 h with a decay after 24 h and 48 h post-transfection, which is emphasized in the strong red fluorescence shift (Fig. 7a4). The difference between both nanoparticles can be further seen in the fluorescence microscopy images (Fig. 8a, b) with highest mCherry protein expression resulting in red fluorescence signal for mRNA:LPNs of ratio 1:20 and 1:30. Nanoparticle sizes of LPN and CS-PLGA changed in the range of 50–100 nm (see Table 1), the increase in PDI was modest and the tendency of size increase could not be related to the observed transfection efficacy. The mRNA:LNPs_1:20 re smaller in size but efficient in transfection. The more likely reason for the stronger transfection efficacy is the availability of remaining cationic groups at the particle surface at ratios of 1:20 or 1:30, which are important for the interaction with cell and endosomal membranes.

Lipid-coated PLGA nanoparticles show a higher transfection efficiency over chitosan-coated PLGA nanoparticles, which may be due to the high stability of complexed mRNA onto CS-PLGA nanoparticles presumably leading to an incomplete release (seen in the gel, Fig. 5b) within the cytoplasmic compartment. mRNA:LPNs correspondingly show fast transfection kinetics as shown in the agarose gel by using heparin (Fig. 4a) releasing the mRNA after 15 min of incubation. Hence, we can suppose that the LPNs survive the acidic condition of the lysosome and escape this compartment slowly over time causing a transient mRNA translation within the cytoplasm reaching a transfection rate already after 2 h with an increment in time and highest transgene expression rate after 4 h. The fluorescence microscope images further indicate a variation of the red fluorescence intensity of mCherry between different cells of the same image. These differences may reflect different metabolic stages of these cells, leading to differences in particle uptake and protein translation, resp., possibly depending on the cell cycle [40].

The transient course observed within this experiments mirrors the behavior of mRNA as reported by Leonhardt et al. [9], in which the transfection kinetics of pDNA and mRNA delivered by the commercial available transfection reagent Lipofectamine2000^®^ was compared. It was shown that mRNA has a faster onset by reaching its maximum transfection efficiency after 3 h. This observation is predictable as pDNA needs enter the nucleus to cause further protein translation while mRNA’s protein expression takes place within the cytoplasm. A further criteria of a rapid transfection rate might be due to method for mRNA loading as it has been already hypothesized by Su et al. [11] that surface-adsorbed mRNA shows a faster release kinetics then encapsulated mRNA. This coincides with the observation made in this in vitro cell study, as LPNs show a faster and complete release (see Fig. 4) and hence the rapid transfection rate compared with mRNA encapsulated within the used positive control JetPRIME^®^ revealing its maximum transfection rate 24 h post-transfection. In contrary, Zhdanov et al. [24, 25] is working on generic models to predict the impact of nanoparticles as nucleotides carriers and their release kinetics on the translation. However, his theoretical models, e.g. using lipid nanoparticles predict only a minor role for the translation kinetics performed in in vitro assays.

Furthermore, while particle-uptake appeared to be similar for mRNA:LPNs and mRNA:CS-PLGA NPs, protein translation of the LPNs mRNA-delivery system was more efficient. Nevertheless, even the weaker performing CS-PLGA NPs had already shown their potential to deliver nuclease-encoding mRNA in vivo in a transgenic mouse model resulting in an efficient genome editing, indicating that the necessary level of delivery efficiency may vary with the therapeutic application [10]. Similar performance of mRNA depending on the application route was observed further by Pardi et al. [41] using commercial lipid particles from an ionizable cationic lipid, PC, and cholesterol-PEG to deliver mRNA in vivo with a variety of delivery routes. The reporter gene luciferase was used to monitor the time of transgene expression. Intravenous and intratracheal delivery showed shorter expression times, with a half-life of mRNA translation ~ 7 h, in comparison to intramuscular and intradermal with a half-life of mRNA translation > 20 h. Knowing the kinetics of both NP-uptake and protein translation of NP-delivered mRNA is equally crucial to develop such tailor-made precision nanomedicines in future.

### Live cell video microscopy: recording NP uptake and protein translation

**Fig. 9:**
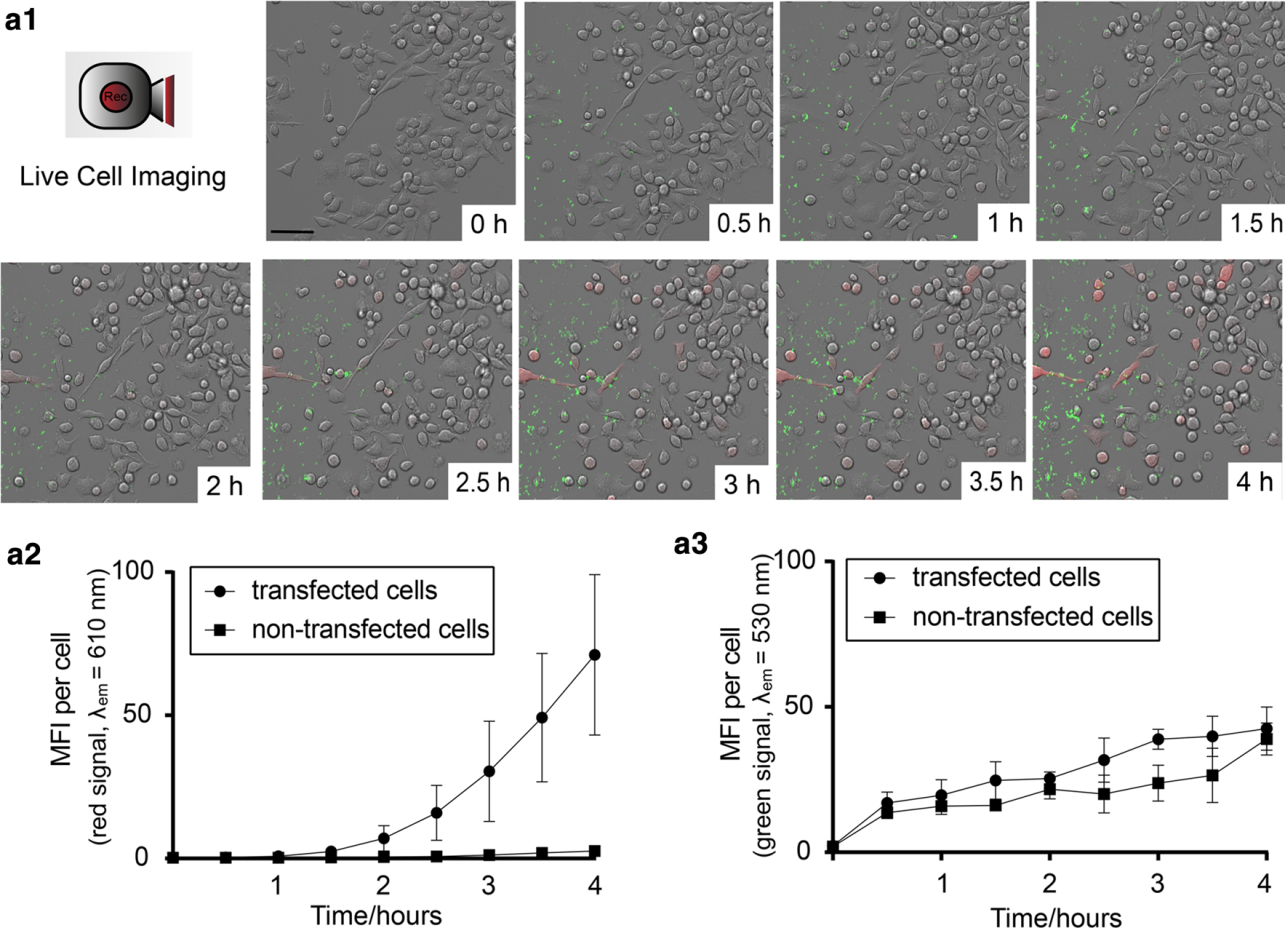
**a1** Representative images depicted from live cell video after nine different time-points. The images are part of a video provided in Additional file 2: Movie S1. DC2.4 cells were incubated with mRNA:FA-LPNs for a complete time duration of 4 h and with an interval of 3 min/image. Green dots on the images represent the fluorescence signal of labeled LPNs, while the cells signaling in red are the ones with successful mCherry expression. Scale bar = 100 µm. **a2** Time-dependent change of the red fluorescence signal resulting from transfected cells and non-transfected, which are correlated with **a3** green fluorescence signal of labeled nanoparticles. The tendency of each single cell being transfected is independent of the NPs uptake, as no significant difference between the uptake-behavior of transfected and non-transfected cells was observable. λ_em_ = peak emission wavelength, *MFI* mean fluorescence intensity (mean ± SD, data from n = 32 fluorescent cells, n = 8 non-fluorescent cells, obtained from 4 independent videos)

In order to simultaneously visualize and quantify both cellular uptake and protein translation NP-mediated mRNA-delivery, we decided to perform live cell video microcopy of this process. DC2.4 cells were exposed to mRNA:FA-LPNs of weight ratio 1:30 and continuously observed over a time-range of 4 h. Figure 9a1 shows representative images depicted from the video after nine different time-points (Additional file 2: Movie S1), with strong particle association to cells seen in green fluorescence and transfected cells signaling in red. Cells showing first a green (= particle binding/uptake) and later a red (= protein translation) fluorescence signal within the 4 h time-frame were counted as transfected cells. Cells showing only a green, but no red fluorescence, were counted as non-transfected cells. As the video analysis shows, particle-cell association starts after 15 min upon exposure while first protein translation signals were recorded after 1 h. After this time-lack, the protein translation (red fluorescence signal) of the transfected cells, as visualized in Fig. 9a2, appeared to increase exponentially until the end of the experiment. As expected, the red fluorescence signal of non-transfected cells remained at the background level. Surprisingly, NP-uptake kinetics (green fluorescence signal) was comparable for both transfected and non-transfected cells with a slow linear increase over time (Fig. 9a3). As live cell video microscopy reveals, some cells start earlier with the transgene expression while others start at later time-point. Quite a few cells do not show transgene expression, even after NPs binding/uptake. This confirms the earlier observed heterogeneity of transgene expression as discussed in the previous section.

### Comparison of transfection efficacy for a non-phagocytic cell line

**Fig. 10:**
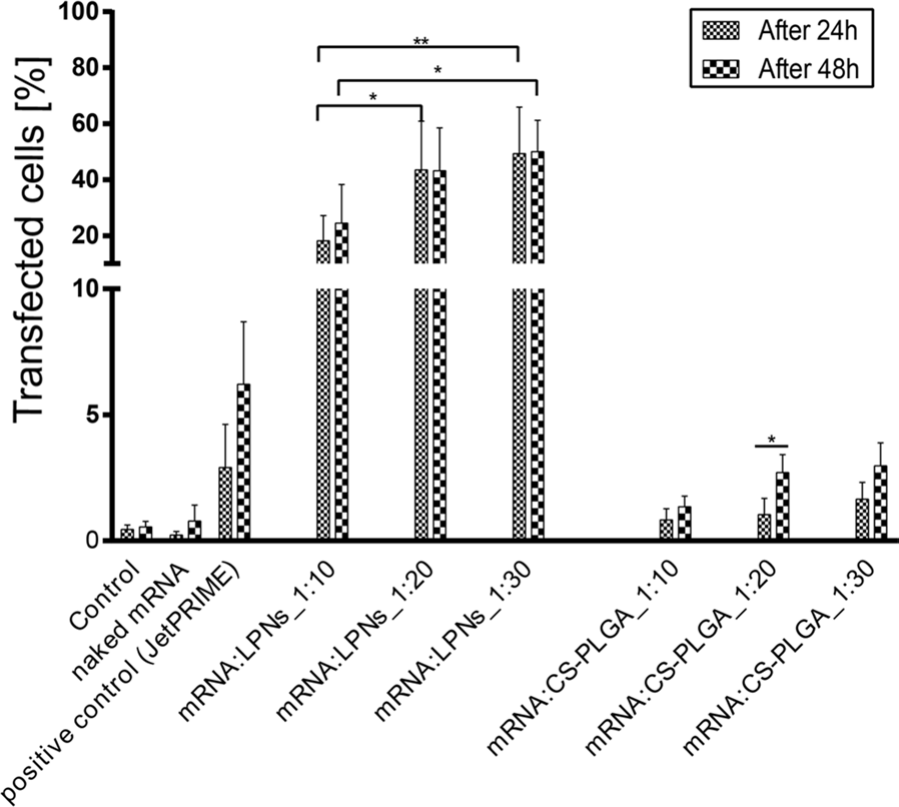
Transfection of non-phagocytic cells, using mRNA:LPNs and mRNA:CS-PLGA at different ratios performed in epithelial A549 cells for 24 h and 48 h post-transfection using flow cytometer. mRNA complexed LPNs reveal a significant higher transgene expression over CS-PLGA NPs. Both NPs show higher transfection rates with higher mRNA:NP ratios and increasing transfection until 48 h post-transfection N = 4, mean ± SD (*p < 0.05 and **p  < 0.01)

To compare the potential of the LPNs to transfect a non-phagocytic cell line by using the same settings as applied for DC2.4 cells, the human alveolar epithelial cell line A549 was chosen, which is a commonly used model for transfection studies. They were grown to ~ 70% confluency in well-plates and then incubated for 4 h with mRNA:LPNs and mRNA:CS-PLGA NPs, resp. The number of transfected cells was counted by flow cytometer 24 and 48 h post-transfection. Similar as for DC2.4 cells, mRNA complexed LPNs revealed a higher efficiency over CS-PLGA NPs with a steadily increasing transfection rate up to ~ 60% for w/w of 1:30 (Fig. 10) with a strong red fluorescence shift (Additional file 1: Figure S6A). Fluorescence confocal images additionally support this observation for mRNA:LPNs (Additional file 1: Figure S6 B). Notably, however, the level of transfected A549 cells still increased up to 48 h post-transfection, whereas transfection of DC2.4 cells started to decrease again after 24 h post-transfection.

## Conclusions

In this study, we have investigated delivery of mRNA into dendritic cells by two different types of cationic nanoparticles. Using fluorescently labeled particles as carriers and mRNA-mCherry as cargo, it was possible to distinguish the different kinetics of NP-uptake and mCherry protein translation. Live cell video microscopy even allowed to follow both processes within the same experiment. The chitosan-PLGA NPs were well internalized by the cells, but relatively inefficient in transfection. Lipid-PLGA hybrid nanoparticles showed superior efficiency transfecting dendritic cells with up to 80% and epithelial cells up 60% transfection rate at concentrations that were not causing any cytotoxic effects. Transgene expression of mRNA:LPNs started in single cells 1 h after particle exposure with an exponential increase during the 4 h of recording. Providing good transfection efficacy and rapid transgene expression, hybrid lipid–polymer nanoparticles, like e.g. the DOTMA LPN delivery system, appear as an interesting platform for mRNA-based therapeutics and vaccination strategies.

## Additional files

**Additional file 1: Figure S1.** Physicochemical characterization for the storage stability of blank LPNs at 4 °C and room temperature (RT) tested over a time-course of 62 days post-preparation **(A)** hydrodynamic size, **(B)** PDI and **(C)** ζ-potential. Colloidal properties reveal stability of LPNs for all tested time-points and temperatures. *N* = 3, mean ± SD. **Figure S2.** (**A1, A2**) Physicochemical characteristics of blank LPNs and (**B1, B2**) blank CS-PLGA NPs tested under different physiological conditions using HBSS buffer, cell culture medium DMEM with and without 10% FCS following 2 h, 4 h and 24 h of incubation. While LPNs show only a significant change in colloidal properties after incubation in DMEM plus 10% FCS, CS-PLGA NPs elicit a significant difference in colloidal parameter for all tested buffers compared with untreated samples. However, the observed size changes are immediate but not increasing within the 24 h of observation. *N* = 3, mean ± SD. **Figure S3.** Representative dot plots and appropriate gating strategy for cytotoxicity assay in DC2.4 cells using blank LPNs and CS-PLGA NPs indicate a fluorescence shift and hence higher cytotoxicity for particles of higher concentrations (160 μg/mL). **Figure S4.** Summary of physicochemical properties for fluoresceinamine labeled blank and labeled mRNA complexed nanoparticles wit mRNA:NPs w/w ratio of 1:10, 1:20 and 1:30. **(A)** Indicates the hydrodynamic size, **(B)** PDI and **(C)** ζ-potential. *N* = 4, mean ± SD. **Figure S5.** Representative dot plots and corresponding gating strategy for the transfection studies in DC2.4 cells using mRNA:LPNs and mRNA:CS-PLGA NPs with JetPRIME^®^ as the positive control, untreated and naked mRNA as negative control. Fluorescence shift reveals cells with mRNA-mCherry transgene expression. **Figure S6.** (**A**) Representative graphs obtained 24 h and 48 h post-transfection of A549 cells with mRNA:LPNs and mRNA:CS-PLGA NPs at different mRNA:NPs weight ratios using flow cytometry. **(B)** Representative confocal images of A549 cells 48 h post-transfection using mRNA:LPNs, JetPRIME^®^ as positive control, naked mRNA as negative control. Red fluorescence reveals cells successfully transfected while their morphology remains consistent with non-transfected cells (green: cell membrane; blue: cell nucleus; scale bar 50 μm).

**Additional file 2: Movie S1.** The movie displays the interaction of fluorescence labeled and mRNA complexed LPNs (weight ratio of 1:30) with DC2.4 cells with the subsequent mCherry protein translation signaling in red fluorescence.

## Abbreviations

DOTMA: 1, 2-di-*O*-octadecenyl-3-trimethylammonium propane
PLGA: poly (lactic-*co*-glycolic acid)
NPs: nanoparticles
LPNs: lipid-PLGA NPs
CS-PLGA NPs: chitosan-PLGA NPs
FA-LPNs: fluoresceinamine labeled lipid-PLGA NPs
CS-FA-PLGA NPs: fluoresceinamine labeled chitosan-PLGA NPs
mRNA:LPNs: mRNA-loaded lipid-PLGA NPs
mRNA:CS-PLGA NPs: mRNA-loaded chitosan-PLGA NPs
mRNA:FA-LPNs: mRNA-loaded fluoresceinamine labeled lipid-PLGA NPs
mRNA:CS-FA-PLGA NPs: mRNA-loaded fluoresceinamine labeled chitosan-PLGA NPs
