# Supplementary material for: Kinetics of mRNA delivery and protein translation in dendritic cells using lipid-coated PLGA nanoparticles
Source: J Nanobiotechnology. 2018 Sep 19;16:72. doi: 10.1186/s12951-018-0401-y (PMC6145106; doi:10.1186/s12951-018-0401-y)
Supplement: Supplementary file 1 — Additional file 1: Figure S1. Physicochemical characterization for the storage stability of blank LPNs at 4 °C and room temperature (RT) tested over a time-course of 62 days post-preparation (A) hydrodynamic size, (B) PDI and (C) ζ-potential. Colloidal properties reveal stability of LPNs for all tested time-points and temperatures. N = 3, mean ± SD. Figure S2. (A1, A2) Physicochemical characteristics of blank LPNs and (B1, B2) blank CS-PLGA NPs tested under different physiological conditions using HBSS buffer, cell culture medium DMEM with and without 10% FCS following 2 h, 4 h and 24 h of incubation. While LPNs show only a significant change in colloidal properties after incubation in DMEM plus 10% FCS, CS-PLGA NPs elicit a significant difference in colloidal parameter for all tested buffers compared with untreated samples. However, the observed size changes are immediate but not increasing within the 24 h of observation. N = 3, mean ± SD. Figure S3. Representative dot plots and appropriate gating strategy for cytotoxicity assay in DC2.4 cells using blank LPNs and CS-PLGA NPs indicate a fluorescence shift and hence higher cytotoxicity for particles of higher concentrations (160 μg/mL). Figure S4. Summary of physicochemical properties for fluoresceinamine labeled blank and labeled mRNA complexed nanoparticles wit mRNA:NPs w/w ratio of 1:10, 1:20 and 1:30. (A) Indicates the hydrodynamic size, (B) PDI and (C) ζ-potential. N = 4, mean ± SD. Figure S5. Representative dot plots and corresponding gating strategy for the transfection studies in DC2.4 cells using mRNA:LPNs and mRNA:CS-PLGA NPs with JetPRIME® as the positive control, untreated and naked mRNA as negative control. Fluorescence shift reveals cells with mRNA-mCherry transgene expression. Figure S6. (A) Representative graphs obtained 24 h and 48 h post-transfection of A549 cells with mRNA:LPNs and mRNA:CS-PLGA NPs at different mRNA:NPs weight ratios using flow cytometry. (B) Representative confocal images of A549 [file 12951_2018_401_MOESM1_ESM.docx]

**Additional file 1**

Kinetics of mRNA delivery and protein translation in dendritic cells using

lipid-coated PLGA nanoparticles

Hanzey Yasar^1,2^, Alexander Biehl^1,3^, Chiara De Rossi^1^, Marcus Koch^4^, Xabi Murgia^1,2^, Brigitta Loretz^1,*^, Claus-Michael Lehr^1,2,3^

^1^ Helmholtz-Institute for Pharmaceutical Research Saarland (HIPS), Helmholtz Center for Infection Research (HZI), Department of Drug Delivery (DDEL), Campus E8.1, 66123 Saarbrücken, Germany; Hanzey.Yasar@helmholtz-hzi.de; Alexander.Biehl@helmholtz-hzi.de; Chiara.DeRossi@helmholtz-hzi.de; Xabier.MurgiaEsteve@helmholtz-hzi.de; Brigitta.Loretz@helmholtz-hzi.de; Claus-Michael.Lehr@helmholtz-hzi.de

^2^ Department of Pharmacy, Saarland University, 66123 Saarbrücken, Germany

^3^ Center for Bioinformatics, Saarland Informatics Campus, Saarland University, 66123 Saarbrücken, Germany

^4^ INM - Leibniz Institute for New Materials, 66123 Saarbrücken, Germany; Marcus.Koch@leibniz-inm.de

***** Correspondence: Brigitta.Loretz@helmholtz-hzi.de; Tel.: +49-681-98806-1030

**Keywords**: mRNA, transfection, gene delivery, chitosan-PLGA, cationic lipid, live cell imaging


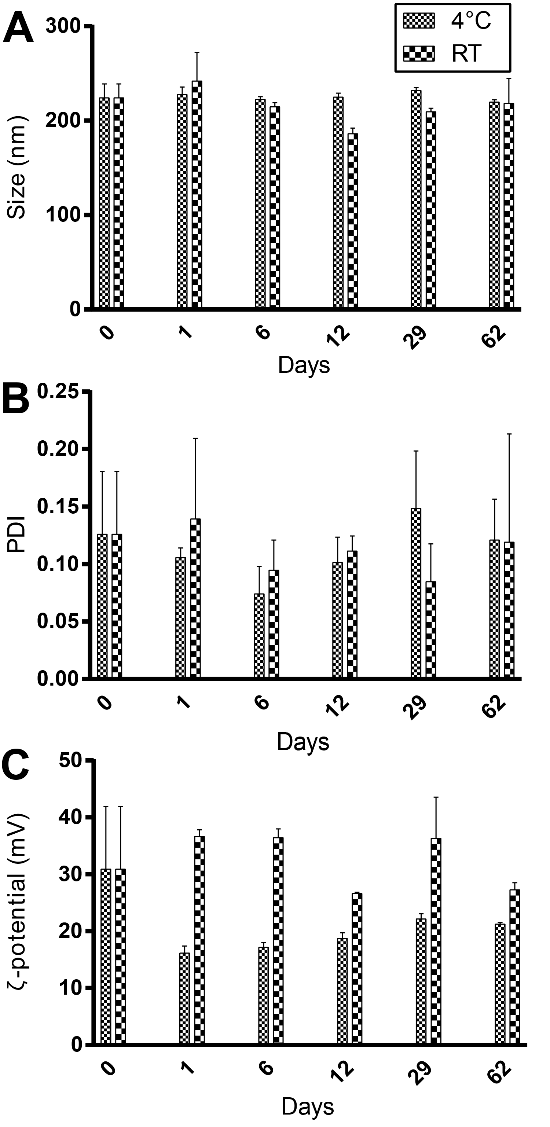


Figure S1: Physicochemical characterization for the storage stability of blank LPNs at 4°C and room temperature (RT) tested over a time-course of 62 days post-preparation **(A)** hydrodynamic size, **(B)** PDI and **(C)** ζ-potential. Colloidal properties reveal stability of LPNs for all tested time-point and temperatures. *N* = 3, mean ± SD.


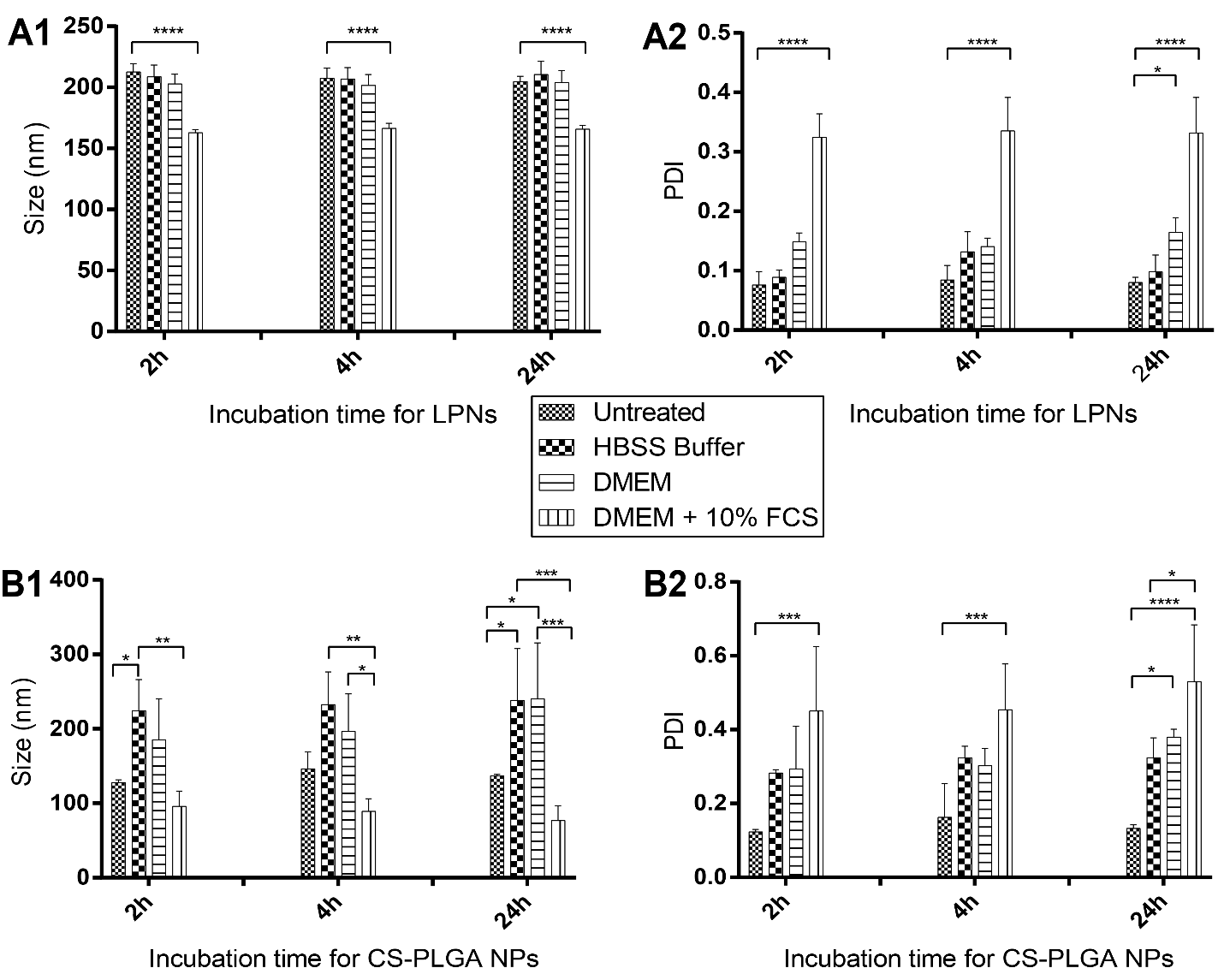


Figure S2: (**A1,** **A2)** Physicochemical characteristics of blank LPNs and (**B1, B2**) blank CS-PLGA NPs tested under different physiological conditions using HBSS buffer, cell culture medium DMEM with and without 10% FCS following 2 h, 4 h and 24 h of incubation. While LPNs show only a significant change in colloidal properties after incubation in DMEM plus 10% FCS, CS-PLGA NPs elicit a significant difference in colloidal parameter for all tested buffers compared with untreated samples. However, the observed size changes are immediate but not increasing within the 24 h of observation. *N* = 3, mean ± SD.


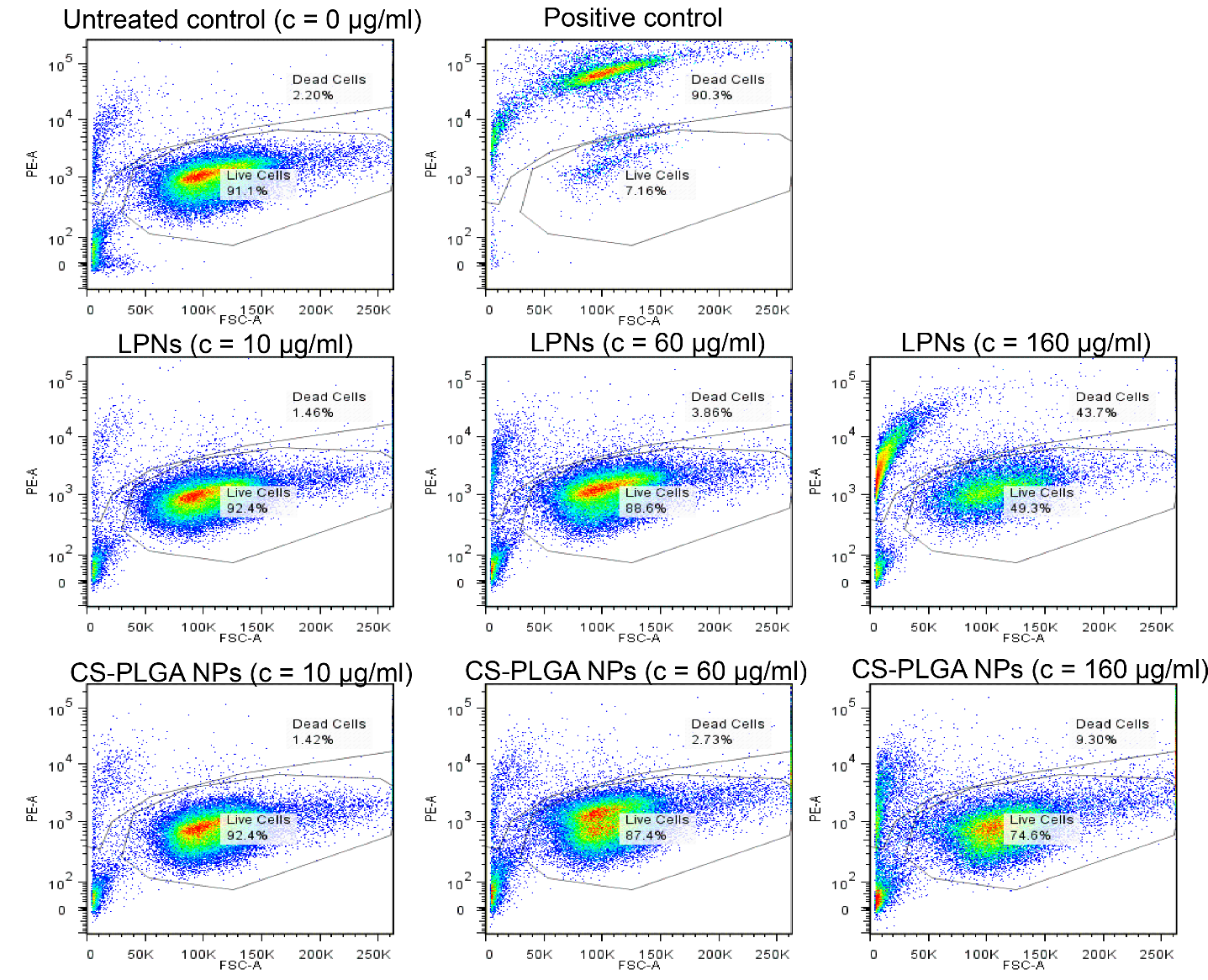


Figure S3: Representative dot plots and appropriate gating strategy for cytotoxicity assay in DC2.4 cells using blank LPNs and CS-PLGA NPs indicate a fluorescence shift and hence higher cytotoxicity for particles of higher concentrations (160 μg/mL).


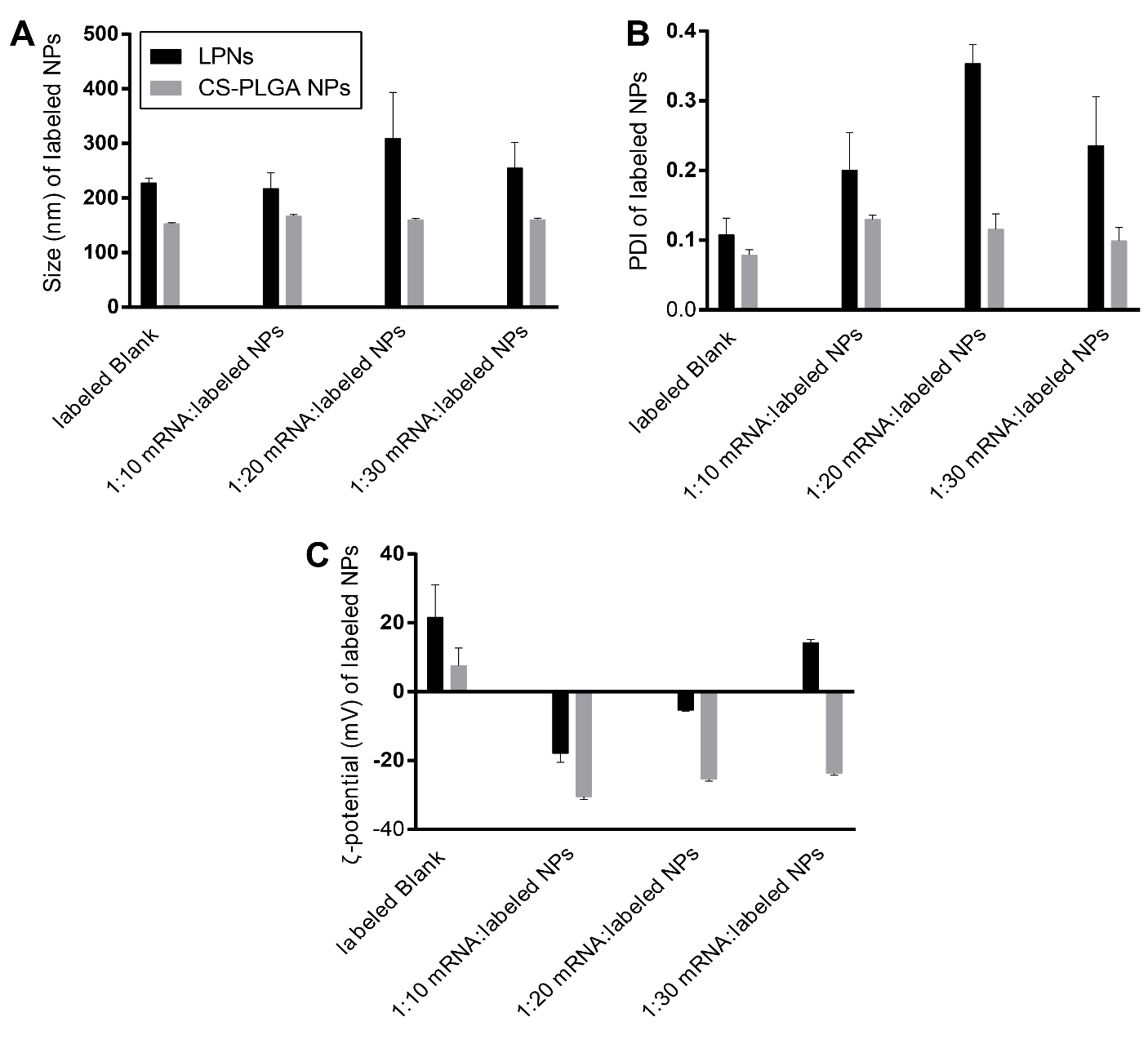


Figure S4: Summary of physicochemical properties for fluoresceinamine labeled blank and labeled mRNA complexed nanoparticles wit mRNA:NPs w/w ratio of 1:10, 1:20 and 1:30. **(A)** Indicates the hydrodynamic size, **(B)** PDI and **(C)** ζ-potential. *N* = 4, mean ± SD.


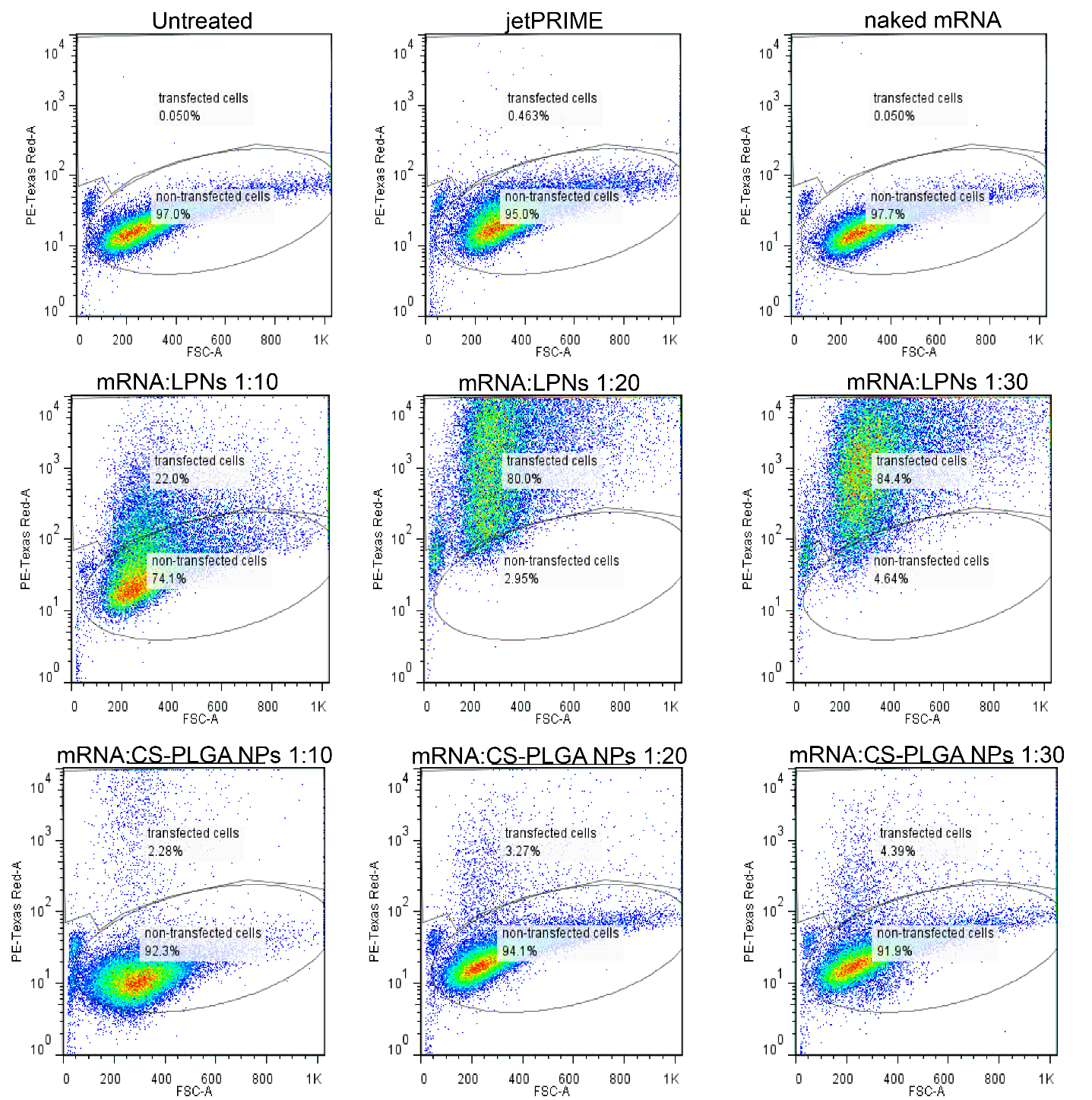


Figure S5: Representative dot plots and corrosponding gating strategy for the transfection studies in DC2.4 cells using mRNA:LPNs and mRNA:CS-PLGA NPs with JetPRIME® as the positive control, untreated and naked mRNA as negative control. Fluorescence shift reveals cells with mRNA-mCherry transgene expression.


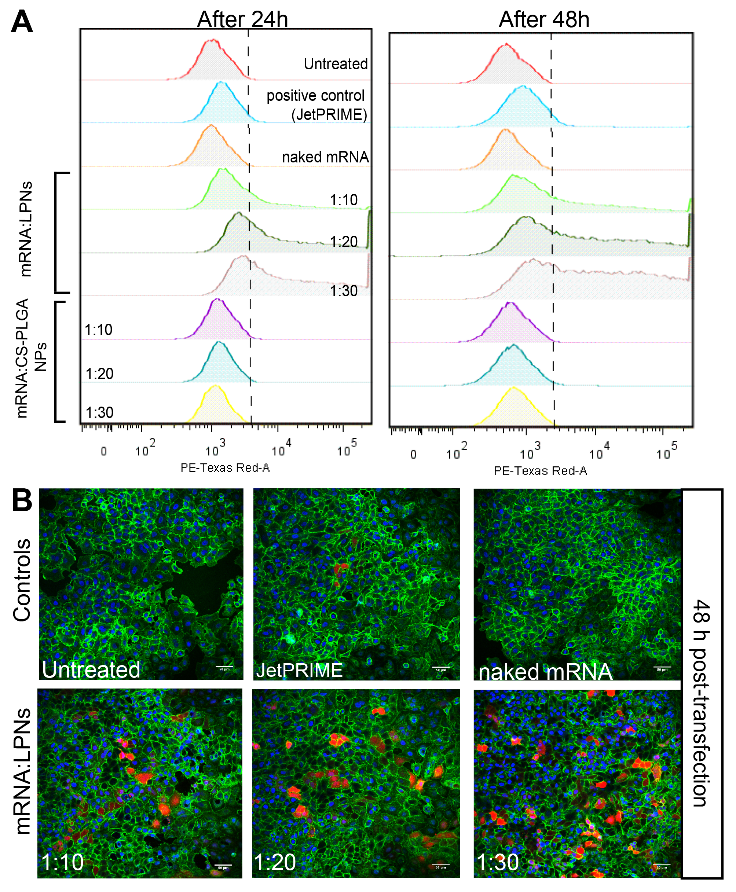


Figure S6: (**A**) Representative graphs obtained 24 h and 48 h post-transfection of A549 cells with mRNA:LPNs and mRNA:CS-PLGA NPs at different mRNA:NPs weight ratios using flow cytometry. **(B)** Representative confocal images of A549 cells 48 h post-transfection using mRNA:LPNs, JetPRIME^®^ as positive control, naked mRNA as negative control. Red fluorescence reveals cells successfully transfected while their morphology remains consistent with non-transfected cells (green: cell membrane; blue: cell nucleus; scale bar 50 μm).
